# Topical Medication Therapy for Glaucoma and Ocular Hypertension

**DOI:** 10.3389/fphar.2021.749858

**Published:** 2021-12-01

**Authors:** Tao Wang, Linlin Cao, Qikun Jiang, Tianhong Zhang

**Affiliations:** ^1^ Department of Pharmaceutics, Wuya College of Innovation, Shenyang Pharmaceutical University, Shenyang, China; ^2^ Department of Pharmaceutics, The Second Hospital of Dalian Medical University, Dalian, China

**Keywords:** glaucoma, monotherapy, fixed combination therapy, non-fixed combination therapy, intraocular pressure, adverse effects

## Abstract

Glaucoma is one of the most common causes of blindness, thus seriously affecting people’s health and quality of life. The topical medical therapy is as the first line treatment in the management of glaucoma since it is inexpensive, convenient, effective, and safe. This review summarizes and compares extensive clinical trials on the topical medications for the treatment of glaucoma, including topical monotherapy agents, topical fixed-combination agents, topical non-fixed combination agents, and their composition, mechanism of action, efficacy, and adverse effects, which will provide reference for optimal choice of clinical medication. Fixed-combination therapeutics offer greater efficacy, reliable security, clinical compliance, and tolerance than non-fixed combination agents and monotherapy agents, which will become a prefer option for the treatment of glaucoma. Meanwhile, we also discuss new trends in the field of new fixed combinations of medications, which may better control IOP and treat glaucoma.

## 1 Introduction

Glaucoma is a neurodegenerative ophthalmologic disease characterized by the progressive degeneration of the retinal ganglion cells and axonal death, resulting in an irreversible blindness (Marshall et al., 2018; Garcia-Medina et al., 2020). Glaucoma is commonly related to high intraocular pressure (IOP) with a subsequent injury of the optic nerve, and eventually blindness (Goel et al., 2010). The normal physiological IOP is 5–20 mmHg, which in turn depends on the adjustment of production and absorption of the aqueous humor (AH) (Goel et al., 2010; Skrzypecki et al., 2018). The ciliary muscle produces the AH that leaves the eye by passive flows through two pathways: the conventional (trabecular meshwork, TM) and unconventional (uveoscleral) pathway (Huang et al., 2018). Normal tension glaucoma (NTG) is characterized by a IOP usually below 21 mmHg, while the primary open-angle glaucoma (POAG) has a IOP of over 22 mmHg (Razeghinejad and Lee, 2019), the latter being the main and modifiable risk factor for glaucoma and its progression (Fogagnolo and Rossetti, 2011; Jayaram, 2020). Ocular hypertension (OHT) is defined as a IOP over 21 mmHg without the damage of the optic disk and view field (Chamard et al., 2020), but it is most likely to develop into open-angle glaucoma (OAG) (Kass et al., 2021).

Currently, the treatments to cure glaucoma mainly consist of surgical intervention and topical medication therapy (Guglielmi et al., 2019; Webb, 2021). Surgical intervention is required when a patient’s visual independence is at risk (Lusthaus and Goldberg, 2019). The risks associated with the surgical procedure and the postoperative recovery are complicated with each patient and the cost is relatively expensive (Razeghinejad and Lee, 2019). Therefore, topical medication therapy with reliable effectiveness and safety is often as the preferred approach in the management of glaucoma (Conlon et al., 2017; Guglielmi et al., 2019; Webb, 2021). The classes of topical monotherapy medications to cure glaucoma include prostaglandin analogs, rho-kinase inhibitors, β-adrenergic blocking agents, α-2 adrenergic agonists, and carbonic anhydrase inhibitors, but they are often inadequate to keep IOP under control (Schehlein et al., 2017). Therefore, the combination of multiple medications is significantly necessary for an adequate control of IOP.

The lowering effect of IOP by the fixed combination and non-fixed combination medications are similar and both the combinations are superior to their constituent agents. Importantly, the fixed combination and non-fixed combination medications have reliable security with no additional new side effects comparing to monotherapy agents. Fixed combination medications have a greater convenience, compliance, and tolerance than non-fixed combination medications, since they can decrease dosing frequency and eliminate the washout effect by decreasing the number of instilled drops, the total number of bottles of medication in use, and the exposure to preservatives (Babic, 2015).

**FIGURE 1:**
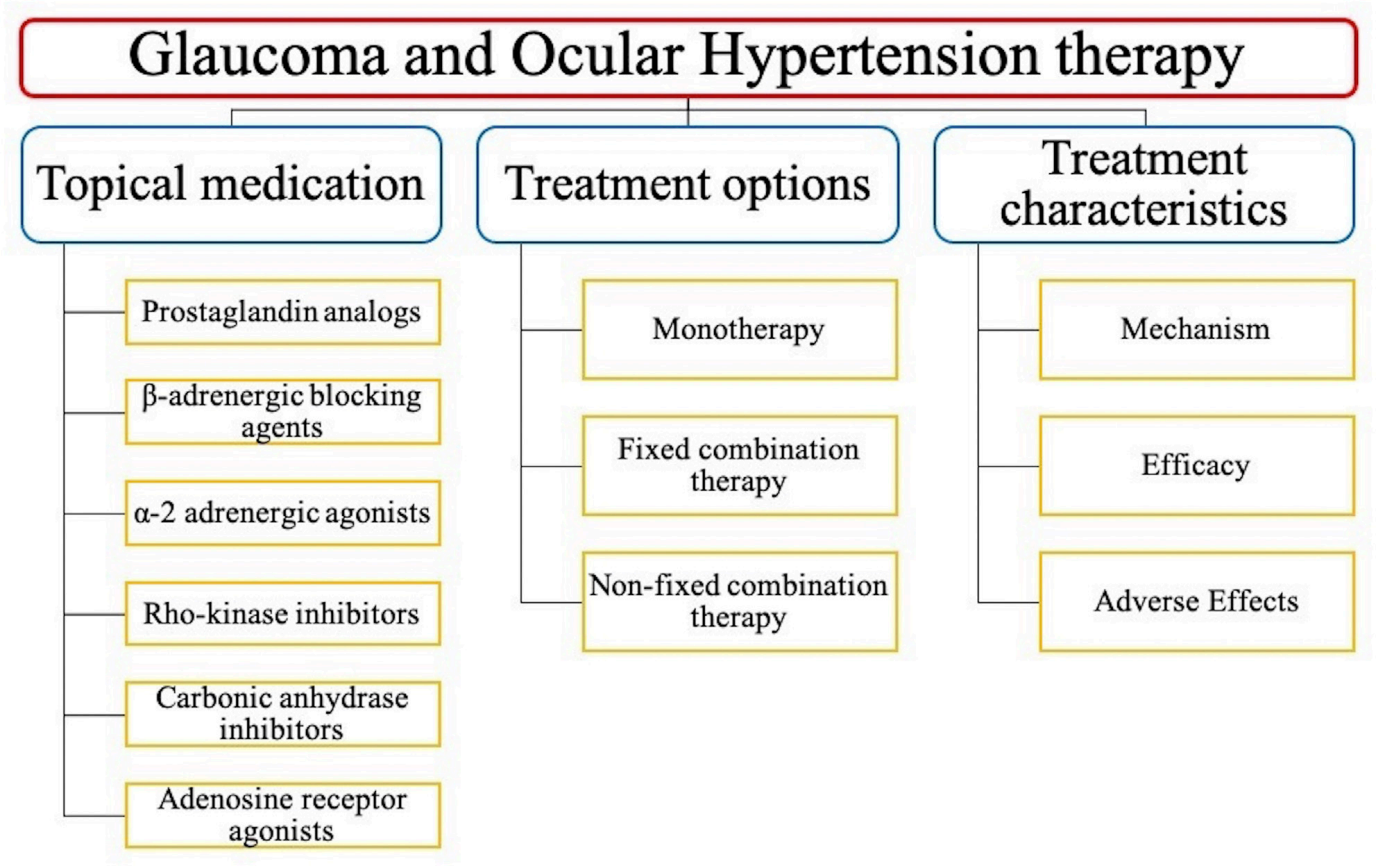
The structure of this review.

This review collects and compares extensive clinical trials on the topical anti-glaucoma medications, and summarized their mechanism of action, efficacy, adverse effects (AEs), and safety on Tables 1–6. And the structure of this review is shown on Figure 1.

**TABLE 1:** The class and list of Prostaglandin Analogs.

Mode of action	Mechanism	Drug	IOP reduction	Current market status	Usage, dosage	Side effects (ocular)
F2 receptor agonists	Increasing uveoscleral outflow	Latanoprost	25–33%	On the market	Once daily	Conjunctival hyperaemia; Eyelash lengthening; Increased periocular and iris pigmentation; Prostaglandin-associated; Periorbitopathy
		Bimatoprost				
		Travoprost				
		Tafluprost				
E2-EP2 receptor dual agonists	Increasing uveoscleral outflow	Omidenepag isopropyl	10–15%	On the market	Once daily	
		Aganepag isopropyl	25–33%	Phase II		
		AGN-210669	—			
E2-EP3-F2-receptor three agonists	Increasing uveoscleral outflow	Sepetaprost	27–30%	Phase II	Once daily	
Nitric-oxide donating F2 receptor agonists	Increasing uveoscleral outflow; Increasing trabecular outflow	Latanoprostene bunod NCX 470	32–34%	On the market Phase III	Once daily	

**TABLE 2:** The class and list of Rho-kinase inhibitors.

Drug	IOP reduction	Current market status	Usage, dosage	Mechanism	Side effects (ocular)
Ripasudil	16–20%	On the market	Twice daily	Increasing trabecular outflow; Decreasing episcleral venous pressure; Reducing aqueous humour formation	Conjunctival hyperemia; Conjunctival hemorrhage; Cornea verticillata; Eye pruritus; Instillation site pain; Increased lacrimation
Netarsudil		On the market	Once daily		
RKI-983/SNJ-1656/Y-39983	—	Phase II	Twice daily		
AMA-0076/PHP-201	—	Phase II	Two or three times daily		
Y-27632	—	Preclinical	—		

**TABLE 3:** The class and list of β blocking agents.

Mode of action	Drug	IOP reduction	Current market status	Usage, dosage	Mechanism	Side effects (ocular)	Side effects (systematic)
Nonselective β blocking agents	Timolol	27–35%	On the market	Twice daily	Reducing aqueous humour formation	Conjunctival hyperemia; Ocular surface discomfort; Reduction in tear flow	Bradycardia; Heart block; Arrhythmia; Bronchospasm; Worsening of underlying asthma or chronic obstructive pulmonary disease
	Carteolol						
Selective β-1 blocking agents	Betaxolol	18–26%					
Selective β-2 blocking agents	Bamosiran	—	Phase II	Once daily			

**TABLE 4:** The class and list of other topical glaucoma monotherapy.

Class	Drug	IOP reduction	Current market status	Usage, dosage	Mechanism	Side effects (ocular)	Side effects (systematic)
α-2 adrenergic agonists	Brimonidine tartrate	20–27%	On the market	Three times daily	Increasing uveoscleral and trabecular outflow; Reducing aqueous humour formation	Blepharitis; Blepharoconjunctivitis; Conjunctivitis; Conjunctival follicles; Mild hyperemia; Staining of the cornea; Blurred vision; Foreign body sensation	—
Carbonic anhydrase inhibitors	Systemic inhibitors: acetazolamide, methazolamide, dichlorophenamide	25–30%	On the market	Twice daily	Reducing aqueous humour formation	—	Numbness and tingling of extremities; Metallic taste; Depression; Fatigue; Malaise; Weight loss; Decreased libido; Gastrointestinal irritation; Metabolic acidosis; Renal calculi; Transient myopia
	Topical inhibitors: Dorzolamide, Brinzolamide	15–25%	On the market	Two or three times daily	Reducing aqueous humour formation	Stinging; Burning or reddening of the eye; Blurred vision; Pruritus; Bitter taste	—
Adenosine receptor agonists	Trabodenoson	—	Discontinued	Twice daily	Increasing trabecular outflow	Eye pain; Conjunctival; Ocular hyperemia; Excoriation	Headache; Back pain; Dermatitis

**TABLE 5:** The class and list of fixed-combination medications.

Classes	Medications	IOP reduction	Current market status	Usage, dosage	Mechanism
Prostaglandin analogs and β-blockers	Latanoprost/Timolol	32–38%	On the market	Once daily	Increasing uveoscleral outflow; Reducing aqueous humour formation
	Bimatoprost/Timolol				
	Travoprost/Timolol				
	Tafluprost/Timolol				
	Latanoprost/carteolol				
Prostaglandin analogs and Rho-kinase inhibitors	Latanoprost/Netarsudil	31–37%	On the market	Once daily	Increasing uveoscleral outflow; Reducing aqueous humour formation; Increasing trabecular outflow; Reducing episcleral venous pressure
Prostaglandin analogs and α-2 adrenergic agonists	Bimatoprost/Brimonidine	25–33%	Phase II	Once daily	Increasing uveoscleral outflow; Reducing aqueous humour formation
Prostaglandin analogs and Carbonic anhydrase inhibitor	Travoprost/Brinzolamide	25–33%	Phase III	Twice daily	Increasing uveoscleral outflow; Reducing aqueous humour formation
	Latanoprost/Dorzolamide	—	Phase II		
β-blockers and α-2 adrenergic agonists	Timolol/Brimonidine	28–34%	On the market	Twice daily	Increasing uveoscleral and trabecular outflow; Reducing aqueous humour formation
β-blockers and Carbonic anhydrase inhibitors	Timolol/Brinzolamide	28–35%	On the market	Twice daily	Reducing aqueous humour formation
	Timolol/Dorzolamide	29–34%			
α-2 adrenergic agonists and Carbonic anhydrase inhibitors	Brinzolamide/Brimonidine	21–35%	On the market	Three times daily	Increasing uveoscleral outflow; Reducing aqueous humour formation
α-2 adrenergic agonists and Rho-kinase inhibitors	Ripasudil/Brimonidine	—	Phase III	—	Increasing uveoscleral outflow; Reducing aqueous humour formation; Increasing trabecular outflow; Reducing episcleral venous pressure
Prostaglandin analogs and β-blockers and α-2 agonists	Bimatoprost/Timolol/Brimonidine	—	Phase III	Twice daily	Increasing uveoscleral outflow; Reducing aqueous humour formation
β-blockers and α-2 agonists and Carbonic anhydrase inhibitors	Dorzolamide/Timolol/Brimonidine	—	On the market	Twice daily	Increasing uveoscleral outflow; Reducing aqueous humour formation

**TABLE 6:** A summary of clinical trials comparing different fixed combination and non-fixed combination agents.

First author/Year/References	Study design	Evaluation period	Diagnosis	Glaucoma medications	Mean baseline IOP (mmHg)	Post treatment IOP (mmHg)	Adverse effects	Comments
Suzuki et al. (2018)	prospective, observer-masked, randomized study	28 days	POAG	BiT-FC (18); LT-FC (14); TrT-FC (18)	13.5 ± 4.2 mmHg	Mean 24-h IOP	—	All three fixed combinations effectively controlled IOP for 24-h and had a similar effect on diurnal and nocturnal IOP variations
						BiT-FC: 14.6 ± 2.9 mmHg		
						LT-FC: 14.1 ± 3.7 mmHg		
						TrT-FC:15.8 ± 2.0 mmHg		
						Mean diurnal IOP variation		
						BiT-FC: 4.6 ± 2.3 mmHg		
						LT-FC: 5.8 ± 2.4 mmHg		
						TrT-FC: 4.3 ± 1.7 mmHg		
						Mean nocturnal IOP variation		
						BiT-FC: 3.2 ± 2.8 mmHg		
						LT-FC: 2.9 ± 1.9 mmHg		
						TrT-FC: 3.0 ± 1.6 mmHg		
Shoji et al. (2013)	multicenter, prospective, randomized, single-blinded, crossover clinical trial	24 weeks with cross over at 12 weeks	NTG	LT-FC (30); TrT-FC (30)	14.8 ± 3.3 mmHg	Mean 24-h IOP (12 weeks)	Burning eye sensation, Mild superficial punctate keratitis, Skin pigmentation: LT-FC: 5.1, 5.1, 1.7%; TrT-FC: 1.7, 1.7, 5.1%	The additional reduction in IOP was greater with TrT-FC than with LT-FC, and their tolerability profiles were similar
						LT-FC: 13.8 ± 3.9 mmHg		
						TrT-FC: 12.4 ± 2.90 mmHg		
						Mean IOP change (24 weeks)		
						LT-FC: 1.1 ± 1.3 mmHg		
						TrT-FC: 2.4 ± 1.3 mmHg		
Eren et al. (2012)	randomized, double-blind, crossover study	6 weeks	(33) OAG	LT-FC; DzT-FC	25.09 ± 2.8 mmHg	Mean diurnal IOP	Bitter taste, Irritation and stinging, Conjunctival hyperemia, Superficial punctate keratitis (case): LT-FC: 0, 1, 2, 1; DzT-FC: 17, 7, 1, 2	Mean diurnal IOP and peak IOP were lower with LT-FC than with DzT-FC. DzT-FC group had significant side effects of stinging and bitter taste
						LT-FC:16.3 mmHg		
						DzT-FC: 17.3 mmHg		
						The peak IOP		
						LT-FC: 18.5 mmHg		
						DzT-FC: 19.9 mmHg		
						Mean diurnal range		
						LT-FC: 4.4 ± 2.2 mmHg		
						DzT-FC: 4.6 ± 2.2 mmHg		
Hommer et al. (2012)	randomized, double-masked, 2-way crossover design	6 weeks	(18) OAG	LT-FC	25.3 ± 2.8 mmHg	The percent of IOP reduction	—	LT-FC and BrT-FC had equally effective in reducing IOP.
			OHT	BrT-FC		LT-FC: 35.0–10.0%		
						BrT-FC: 33.6–8.8%		
Shim et al. (2014)	prospective, clinical study	24 h	healthy subjects	LT-FC (30); BiT-FC (28)	LT-FC: 11.18 ± 3.27 mmHg (treated eye), 11.36 ± 3.07 mmHg (untreated eye); BiT-FC: 13.00 ± 2.12 mmHg (treated eye), 12.60 ± 2.09 mmHg (untreated eye)	The largest difference in IOP (between treated and untreated eyes)	Cojunctival hyperemia, Foreign-body sensation, Superficial punctate epitheliopathy	LT-FC and BiT-FC provided a significant reduction in IOP from baseline with no significantly difference in side effects
						LT-FC: 1.93 mmHg (10 h after instillation)	LT-FC: 25, 17.9, 10.7%	
						BiT-FC: 1.67 mmHg (8 h after instillation)	BiT-FC: 10, 20, 13.3%	
Konstas et al. (2014)	prospective, observer-masked, active controlled, cross-over, comparison study	3 months	OAG	LT-FC (20); PF-TrT-FC (22)	21.5 ± 1.6 mmHg	Mean 24-h IOP	Tear film break-up time, Corneal stain, Schirmer I test	The mean 24-h IOP lowering of TrT-FC was statistically more significant compared to LT-FC in patients
						LT-FC: 19.3 ± 2.3 mmHg	LT-FC: 4.65 s, 1.8 s, 9.2 mm	
						PF-TrT-FC: 18.9 ± 2.2 mmHg	PF-TrT-FC: 5.15 s, 1.5 s, 9.9 mm	
Zhao et al. (2011)	randomized, open-label, parallel-group, noninferiority study	8 weeks	OAG, OHT	LT-FC (125); LT-nFC (125)	LT-FC: 25.8 mmHg; LT-nFC: 26.0 mmHg	Mean diurnal IOP changes	The incidence of AEs: LT-FC: 11.2%, LT-nFC: 6.5%; Conjunctival hyperemia: LT-FC: 7.2%, LT-nFC: 4.8%	The fixed-combination of latanoprost and timolol was as effective as the non-fixed combination
						LT-FC: −8.6 mmHg		
						LT-nFC: −8.9 mmHg		
Higginbotham et al. (2010)	randomized, double-masked, parallel-group study	12 weeks	OAG, OHT	LT-FC (129); Latanoprost (134); Timolol (131)	LT-FC: 28.0 (2.2) mmHg; Latanoprost: 28.2 (2.2) mmHg; Timolol: 28.1 (2.3) mmHg	Mean diurnal IOP (6 weeks, 12 weeks)	The incidence of ocular-related AEs: LT-FC: 17.8%, Latanoprost: 23.9%, Timolol: 10.7%; Conjunctival hyperemia: LT-FC: 7.0%, Latanoprost: 10.4%, Timolol: 3.1%	LT-FC therapy is as safe and effective in lowering IOP in patients with either ocular hypertension or glaucoma as monotherapy with latanoprost or timolol
						LT-FC: 17.9 (3.3), 17.8 (3.5) mmHg		
						Latanoprost: 18.9 (2.9), 19.3 (3.4) mmHg		
						Timolol: 20.9 (3.7), 20.9 (3.5) mmHg		
						Mean diurnal IOP reductions from baseline (12 weeks)		
						LT-FC: 73.5%		
						Latanoprost: 57.5%		
						Timolol: 32.8%		
Asrani et al. (2019)	randomized, double masked, phase 3 clinical trial	3 months	OAG, OHT	NL-FC (238); Latanoprost (236); Netarsudil (244)	22.4–24.8 mmHg	Mean percent of diurnal IOP changes: NL-FC: −33.7%, Latanoprost: −27.6%, Netarsudil: −22.8%	Conjunctival hyperemia	NL-FC provided clinically and statistically significantly greater IOP-lowering effect than monotherapy. The incidence rate of conjunctival hyperemia significant increases, but it is acceptable
							NL-FC: 63.0%	
							Latanoprost: 62.5%	
							Netarsudil: 64.1%	
							Conjunctival hyperemia led to treatment discontinuation	
							NL-FC: 7.1%	
							Latanoprost: 0%	
							Netarsudil: 4.9%	
Asrani et al. (2020)	Phase 3 clinical trial	3 months	OAG, OHT	NL-FC (483); Latanoprost (486); Netarsudil (499)	23.6 mmHg; 23.5 mmHg; 23.6 mmHg	Mean diurnal IOP: NL-FC: 15.8 mmHg, Latanoprost: 17.3 mmHg, Netarsudil: 18.4 mmHg; The percent of IOP reduction >40%: NL-FC: 30.9%, Latanoprost: 8.5%, Netarsudil: 5.9%	Conjunctival hyperemia, Cornea verticillate, Junctival hemorrhage: NL-FC: 58.7,15.4, 10.8%; Latanoprost: 22.1, 0, 1.0%; Netarsudil: 47.0, 11.6, 14.5%	
Konstas et al. (2013b)	prospective, observer-masked, crossover, comparison protocol	3 months	high-pressure exfoliation syndrome (glaucoma)	BiT-FC (21); latanoprost (20)	31.1 mmHg	The mean 24-h IOP reduction: BiT-FC: 12.2 mmHg (39.2%), Latanoprost: 9.9 mmHg (31.9%); The mean 24-h IOP fluctuation: BiT-FC: 3.8 mmHg, Latanoprost: 4.2 mmHg	—	Comparing to latanoprost, BiT-FC had a better IOP-lowering effect. However, both medications had no difference in mean 24-h IOP fluctuation
Lewis et al. (2010)	identical, double-masked, parallel studies	12 months	OHT	BiT-FC (533); Bimatoprost (n = 265); Timolol (n = 263)	23.3–26.2 mmHg	Mean diurnal IOP reduction	The percent of treatment-related AEs	BiT-FC provided statistically significant greater reduction in IOP over the 12-months than bimatoprost and timolol monotherapies. And there is no addition AEs appeared in BiT-FC group
						BiT-FC: 7.7 mmHg	BiT-FC: 48.0%	
						Bimatoprost: 7.6 mmHg	Bimatoprost: 60.0%	
						Timolol: 6.4 mmHg	Timolol: 31.6%	
						The percent of IOP <18 mmHg	Conjunctival hyperemia	
						BiT-FC: 23.3%	BiT-FC: 25.7%	
						Bimatoprost: 18.1%	Bimatoprost: 43.4%	
						Timolol: 8.0%	Timolol: 8.7%	
Pfeiffer et al. (2014)	stratified, double-masked, randomized, multicenter phase III study	3 months	OAG, OHT (prior timolol users)	PF-TfT-FC (95); Timolol (94)	—	The average diurnal IOP change: PF-TfT-FC: −8.55 mmHg (32%)	Conjunctival/ocular hyperemia: PF-TfT-FC: 9.5%, Timolol: 0%	PF-TfT-FC with a substantial and significant IOP reduction effect was superior to monotherapy. No statistically significant differences in the drop discomfort among all groups
						Timolol: −7.35 mmHg (28%)		
			OAG	PF-TfT-FC (188)	—	The average diurnal IOP change: PF-TfT-FC: −8.61 mmHg (33%)	Conjunctival/ocular hyperemia: PF-TfT-FC: 4.8%, Tafluprost: 3.2%	
			OHT (prior prostaglandin analog users)	Tafluprost (187)		Tafluprost: 7.23 mmHg (28%)		
Babic et al. (2013)	randomized, controlled, open-label, prospective study	3 months	POAG, OHT	TrT-FC (30); DzT-FC (26)	25.10 mmHg; 24.23 mmHg	Mean IOP reduction: TrT-FC: 8.96 mmHg (36.28%), DzT-FC: 8.07 mmHg (35.66%)	The most frequent ocular AEs: TrT-FC group: hyperemia (50%), blurred vision and pruritus (6.7%); DzT-FC group: dry eye sensation (30.8%), foreign body sensation (23.1%)	TrT-FC was slightly more effective than DzT-FC in reducing mean diurnal IOP. Both treatments were well tolerated and safe
Macky, (2014)	prospective, randomized, controlled, clinical study	6 months	POAG	BiT-FC (40); TrT-FC (40)	23.00 mmHg; 24.00 mmHg	Mean IOP reduction: BiT-FC: 11.17 mmHg (42.5%), TrT-FC: 7.89 mmHg (33.3%)	Ocular redness: BiT-FC: 10.0%, TrT-FC: 12.5%	BiT-FC can provide more effective IOP reduction than TrT-FC.
Pfeiffer and group, (2011)	double-masked	12 weeks	OAG, OHT	TrBz-nFC (90); TrT-nFC (90)	21.0 ± 2.2 mmHg; 21.2 ± 2.2 mmHg	Mean IOP reduction: TrBz-nFC: 3.2 ± 2.4 mmHg (14.8 ± 10.5%), TrT-nFC: 4.2 ± 2.8 mmHg (19.6 ± 12.7%)	Conjunctival hyperemia: TrBz-nFC: 8.3%, TrT-nFC: 5.4%	Timolol added to travoprost reduced IOP more effectively than brinzolamide added to travoprost
Yamamoto et al. (2016)	randomized, controlled, phase 3 trials	8 weeks	POAG, OHT, NTG	LC-FC (118); Latanoprost (119)	20.1 ± 2.2 mmHg; 20.1 ± 1.9 mmHg	Mean IOP reduction: LC-FC: 2.9 mmHg, Latanoprost: 1.6 mmHg	The incidence of drug-related AEs: LC-FC: 6.8%, Latanoprost: 4.5%	Both LC-FC and LC-nFC had comparable effects and achieved a significantly greater IOP-lowering effect than latanoprost and carteolol
				LC-FC (78); carteolol (78); LC-nFC (37)	19.8 ± 1.7 mmHg; 19.8 ± 2.4 mmHg; 19.7 ± 2.1 mmHg	Mean IOP reduction: LC-FC: 3.5 mmHg, carteolol: 1.6 mmHg, LC-nFC: 3.1 mmHg	The incidence of drug-related AEs: LC-FC: 19.2%, carteolol: 2.6%, LC-nFC: 16.2%	
Kim et al. (2016)	multi-institution, randomized, active-controlled, open-label, parallel-group study	12 weeks	NTG	BrT-FC (48); Timolol (47)	15.2 ± 3.42 mmHg; 14.58 ± 3.05 mmHg	Mean IOP: BrT-FC: 11.87 ± 2.51 mmHg, Timolol: 12.76 ± 3.14 mmHg; The ratio of IOP reduction >20%: BrT-FC: 56%, Timolol: 23.53%	The incidence of ocular AEs: BrT-FC: 18.18%, Timolol: 5.45%; The incidence of systemic AEs: BrT-FC: 1.82%, Timolol: 3.64%	BrT-FC has a superior IOP-lowering effect than timolol in NTG patients, which also has well tolerated and safe
Katz et al. (2012)	prospective, randomized, multicenter, investigator-masked clinical trial	12 weeks	POAG, OHT	BrT-FC (73); Latanoprost (75)	24.7 mmHg; 25.4 mmHg	The mean diurnal IOP: BrT-FC: 17.8 (2.9) mmHg, Latanoprost: 17.9 (3.9) mmHg; The mean change in diurnal IOP: BrT-FC: 7.0 mmHg (27.9%), Latanoprost: 7.5 mmHg (29.7%)	The incidence of AEs: BrT-FC: 21.9%, Latanoprost: 10.7%; Conjunctival hyperemia, Lid Edema: BrT-FC: 11.6%, 5.8%; Latanoprost: 16.0%, 1.3%	BrT-FC and latanoprost have similar efficacy in lowering IOP in patients with glaucoma or OHT over 12 weeks. AEs were more common in BrT-FC group, but none of the adverse events were serious
Gulkilik et al. (2011)	prospective study	4 weeks	POAG	BrT-FC (42); DzT-FC (42)	24.6 mmHg; 24.1 mmHg	The mean diurnal IOP: BrT-FC: 16.9 mmHg, DzT-FC: 17.3 mmHg	The Schirmer scores (tear function tests) (before and after): BrT-FC: 14.1 ± 2.2 and 13.2 ± 3.0 mm, DzT-FC: 13.3 ± 2.8, and 12.3 ± 3.8 mm; The tear break-up time (before and after): BrT-FC: 10.9 ± 1.9 and 9.9 ± 1.9 s, DzT-FC: 10.1 ± 1.8 and 9.1 ± 1.6 s; Burning, Foreign body sensation, Itching: BrT-FC: 19, 12, 14%; DzT-FC: 43, 28, 12%	Both BrT-FC and DzT-FC can effectively lower IOP. The side-effect profile is similar in both groups. BrT-FC reduces lesser occurrence of a burning sensation, which may improve patient compliance. Both groups significantly lower tear secretion and tear break-up time, which may lead to dry eye
Realini et al. (2013)	identical, phase 3, randomized, clinical trials	3 months	OAG, OHT	BzBr-FC (424); Brinzolamide (453); Brimonidine (448)	23.7–27.0 mmHg; 23.9–27.2 mmHg; 23.7–27.2 mmHg	The mean diurnal IOP: BzBr-FC:16.5–20.2 mmHg, Brinzolamide:19.5–21.2 mmHg, Brimonidine:18.0–22.5 mmHg	Vision blurred, Eye irritation, Ocular hyperemia, Dysgeusia, Dry mouth: BzBr-FC: 5.3, 4.1, 2.1, 3.9, 3.0%; Brinzolamide: 6.4, 1.1, 0.7, 8.3, 0.0%; Brimonidine: 0.2, 2.2, 3.3, 0.2, 2.4%	BzBr-FC had significantly superior IOP-lowering activity compared with either brinzolamide or brimonidine alone and a safety profile consistent with that of its individual components
Aung et al. (2014)	Phase 3, randomized, multicenter, double-masked clinical trial	6 months	OAG	BzBr-FC (193); Brinzolamide (192); Brimonidine (175)	25.9 ± 0.19 mmHg; 25.9 ± 0.2 mmHg; 26.0 ± 0.19 mmHg	The mean diurnal IOP reduction (weeek-2, month-3 and month-6): BzBr-FC: 7.6, 7.9, 7.8 mmHg; Brinzolamide; 6.1, 6.5, 6.7 mmHg; Brimonidine: 6.0, 6.4, 6.4 mmHg	The incidence of serious AEs: BzBr-FC: 2.6%, Brinzolamide: 1.0%, Brimonidine: 1.7%; The incidence of discontinuation: BzBr-FC: 9.3%, Brinzolamide: 0.5%, Brimonidine: 7.4%; The incidence of treatment-related AEs: BzBr-FC: 28.5%, Brinzolamide: 11.5%, Brimonidine: 22.9%	BzBr-FC had a significantly greater IOP-lowering effect than either brinzolamide or brimonidine alone and displayed a safety profile consistent with its individual components
Katz et al. (2013)	Phase 3, double-masked, parallel group, multicenter study	3 months	OAG, OHT	BzBr-FC (220); Brinzolamide (220); Brimonidine (220)	26.9–23.2 mmHg; 27.1–23.6 mmHg; 27.0–24.0 mmHg	The mean diurnal IOP reduction: BzBr-FC: 24.1–34.9%, Brinzolamide: 16.9–22.6%, Brimonidine: 14.3–25.8%	Vision blurred, Ocular hyperemia, Dry eye, Dysgeusi: BzBr-FC: 6.1, 3.3, 0.9, 3.7%, Brinzolamide: 6.2, 0.9, 0.9, 6.2%, Brimonidine: 0.5, 4.1, 2.7, 0%	BzBr-FC has significantly superior IOP-lowering activity compared with either brinzolamide alone or brimonidine alone in patients with open-angle glaucoma or ocular hypertension while providing a safety profile consistent with that of its individual components
Seibold et al. (2017)	prospective, multicenter, double-masked, parallel-group clinical trial	24 h	OAG, OHT	BzBr-FC (30); Timolol (30)	The baseline 24 h IOP: 20.2 ± 0.5 mmHg, 19.8 ± 0.6 mmHg; The baseline diurnal IOP: 19.7 ± 0.5 mmHg, 19.3 ± 0.7 mmHg; The baseline nocturnal IOP: 21.8 ± 0.5 mmHg, 21.4 ± 0.6 mmHg	The mean 24 h IOP reduction: BzBr-FC: 2.0 ± 0.3 mmHg, Timolol: 1.2 ± 0.3 mmHg; The mean diurnal IOP change: BzBr-FC: 2.7 ± 0.4 mmHg, Timolol: 2.1 ± 0.43 mmHg; The mean nocturnal IOP change: BzBr-FC: 0.8 ± 0.3 mmHg, Timolol: +0.6 ± 0.2 mmHg	—	Both BzBr-FC and timolol significantly lower IOP during the diurnal period. During the nocturnal period, the effect is lessened but remains significant for BzBr-FC, while timolol fails to reduce IOP overnight
Nguyen et al. (2013)	Phase 3, multicenter, double-masked, parallel-group	3 months	OAG, OHT	BzBr-FC (218); Brinzolamide (229); Brimonidine (232)	24.1–27.2 mmHg; 27.2–24.2 mmHg; 23.7–27.3 mmHg	The mean IOP reduction: BzBr-FC: 5.4–8.4 mmHg, Brinzolamide: 4.2–5.7 mmHg, Brimonidine: 3.1–6.5 mmHg	Blurred vision, eye pruritus, dysgeusia: BzBr-FC: 4.5, 2.3, 4.1%; Brinzolamide: 6.8, 1.3, 10.3%; Brimonidine: 0, 0, 0.4%; Conjunctivitis, dry mouth, eye allergy: BzBr-FC: 1.8, 2.7, 4.5%; Brinzolamide: 0, 0, 0%; Brimonidine: 3.0, 2.1, 0.9%; The rate of discontinued participation: BzBr-FC: 11.3%, Brinzolamide: 2.1%, Brimonidine: 9.4%	BzBr-FC has significantly superior IOP-lowering activity compared with either brinzolamide or brimonidine, while providing a safety profile which is consistent with that of the individual components
Sezgin Akcay et al. (2013)	randomized, double-blinded, active-controlled, parallel-group trial	3 months	OAG, OHT	BzT-FC (57); DzT-FC (57)	24.6–29.9 mmHg	The mean IOP: BzT-FC: 16.26–18.98 mmHg, DzT-FC: 16.35–19.0 mmHg; The mean IOP reductions: BzT-FC: 6.42–9.74 mmHg (26.09–37.46%), DzT-FC: 8.16–12.41 mmHg (31.19–41.44%)	Blurred vision, Ocular irritation, Eye pain, Foreign body sensation: BzT-FC: 33.3, 7.01, 3.5, 5.2%; DzT-FC: 10.5, 33.3, 29.8, 28.07%	DzT-FC and BzT-FC provided comparable IOP-lowering effect, while greater ocular discomfort was happened in DzT-FC group. BzT-FC treatment may provide a better patient experience and improve therapeutic compliance
Hartleben et al. (2017)	multicenter, double-masked, randomized, phase 3 study	12 weeks	POAG, OHT	BiBrT-FC (93); BrT-FC (97)	24.62 ± 2.48 mmHg; 25.12 ± 2.18 mmHg	The mean IOP reductions: BiBrT-FC: 10.03 mmHg, BrT-FC: 9.18 mmHg; The proportion of IOP<13 mmHg: BiBrT-FC: 33.7%, BrT-FC: 14.8%	The incidence of treatment-related AEs: BiBrT-FC: 52.7%, BrT-FC: 27.6%; The percentage of discontinuations: BiBrT-FC: 5.4%, BrT-FC: 4.1%; Conjunctival hyperemia, Eye irritation, Dry eye, Eye pruritus, Somnolence: BiBrT-FC: 23.7, 14.0, 8.6, 6.5, 4.3%; BrT-FC: 3.1, 4.1, 1.0, 2.0, 8.2%	BiBrT-FC had superior ocular hypotensive effects (compared with BrT-FC). Although, the ocular side effects of BiBrT-FC are more serious than BrT-FC, BiBrT-FC offers IOP-lowering benefits that may outweigh the risk
Belfort et al. (2020)	randomized, masked, controlled, phase III study	12 weeks	POAG, OHT	BiBrT-FC (90); BrT-FC (95)	25.4 mmHg; 24.4 mmHg	The mean IOP: BiBrT-FC: 15.0 mmHg, BrT-FC: 16.0 mmHg; The mean IOP reductions: BiBrT-FC: 10.45 mmHg, BrT-FC: 8.28 mmHg	The incidence of treatment-related AEs: BiBrT-FC: 72.2%, BrT-FC: 53.7%; Conjunctival hyperemia, Eye pruritus: BiBrT-FC: 47.8%, 12.2%; BrT-FC: 23.2%, 4.2%	BiBrT-FC provided clinically and statistically significantly superior IOP-lowering efficacy than did DFC with no unexpected AEs or marked worsening of expected AEs arising from the combination of these 3 medications into 1 ophthalmic solution
Fechtner et al. (2011)	prospective, randomized, multicenter, investigator-masked, parallel-group study	12 weeks	OAG	BrT-FC + Latanprost (102); LT-nFC (102)	23.4–23.7 mmHg; 23.0–23.5 mmHg	The mean IOP: 15.1–17.0 mmHg; 16.9–17.7 mmHg	The incidence of treatment-related AEs: BrT-FC + Latanoprost: 9.8%, LT-nFC: 3.9%; The incidence of ocular AEs: BrT-FC + Latanoprost: 8.8%, LT-nFC: 6.9	BrT-FC reduces IOP significantly more effectively than timolol when used as therapy adjunctive to latanoprost. Adjunctive treatment with BrT-FC was also well tolerated
Konstas et al. (2013a)	prospective, observer-masked, active controlled, crossover, comparison	3 months	POAG	BzT-FC + Travoprost (23); BrT-FC + Travoprost (27)	20.1 mmHg	The mean 24 h IOP: BzT-FC + Tr: 17.2 mmHg, BrT-FC + Tr: 18.0 mmHg; The mean 24 h IOP fluctuation: BzT-FC + Tr: 3.6 mmHg, BrT-FC + Tr: 4.3 mmHg	Hypertrichosis, Systemic hypotension, Dry eye sensation	BzT-FC + Travoprost achieves a better mean 24-h IOP control
Konstas et al. (2008)	prospective, observer-masked, placebo controlled, crossover, comparison	3 months	(31) OAG	DzT-FC + Latanoprost; DzT-FC; LT-FC	22.1 mmHg	The mean IOP reduction: DzT-FC + Latanoprost: 5.6 mmHg, DzT-FC: 2.2 mmHg, LT-FC: 2.7 mmHg; The mean 24 h IOP fluctuation: DzT-FC + Latanoprost: 3.6 mmHg, DzT-FC: 4.4 mmHg, LT-FC: 4.1 mmHg	Burning/stinging; Watering; Superficial punctate keratitis	DzT-FC + Latanoprost demonstrates the greatest pressure reduction
Hatanaka et al. (2010)	prospective, open-label, randomized, controlled clinical Trial	4 weeks	OAG	DzT-FC + Latanoprost (49); Latanoprost (49)	15.24 ± 2.84 mmHg; 15.34 ± 2.96 mmHg	The mean IOP: DzT-FC + Latanoprost: 14.44 ± 3.03 mmHg, Latanoprost: 15.60 ± 3.09 mmHg	—	DzT-FC as an adjunct to latanoprost may further enhance pressure reduction
Konstas et al. (2017)	prospective, observer-maske, crossover, comparison	24 h	OAG	PF-DzT-FC + PF-Tafluprost (21); PF-Tafluprost (22)	22.2 ± 2.9 mmHg	The mean 24 h IOP: PF-DzT-FC + PF-Tafluprost: 17.3 ± 2.7 mmHg, PF-Tafluprost: 21.9 ± 3.2 mmHg; The mean Daytime IOP: PF-DzT-FC + PF-Tafluprost: 17.0 mmHg, PF-Tafluprost: 22.3 mmHg; The mean Nighttime IOP: PF-DzT-FC + PF-Tafluprost:17.6 mmHg, PF-Tafluprost: 21.5 mmHg	Corneal stain (van Bijsterveld score), Schirmer test (mm), Break-up time (s): PF-DzT-FC + PF-Tafluprost: 1.7, 8.2, 6.1; PF-Tafluprost: 1.3, 9.1, 6.7; Stinging, Hyperemia, Blurring of vision, Itchiness: PF-DzT-FC + PF-Tafluprost: 20.9, 9.3, 4.6, 2.3%; PF-Tafluprost: 6.9, 11.6, 6.9, 6.9%	The combination of PF-DzT-FC and PF-Tafluprost provided statistically greater 24-h efficacy and improved tolerability
Topouzis et al. (2021)	Phase 4, randomized (1:1), double masked, parallel group trial	6 weeks	OAG	BrBz-FC + PGA (96); PGA (92)	28.8 mmHg; 28.9 mmHg	The mean diurnal IOP change: BrBz-FC + PGA: −5.59 mmHg, PGA: 2.15 mmHg	The incidence of ocular AEs: BrBz-FC + PGA: 21.1%, PGA: 8.7%; Ocular hyperemia, Conjunctival hyperemia, Dry mouth: BrBz-FC + PGA: 5.3, 4.2, 5.3%; PGA: 1.1, 1.1, 0%	BrBz-FC as an adjunct to PGA is a suitable treatment option for patients with open-angle glaucoma or ocular hypertension for whom PGA monotherapy provides insufficient IOP reduction. The safety profile of BrBz-FC + PGA was consistent with the known safety profiles of brinzolamide, brimonidine, and PGAs
Fechtner et al. (2016)	multicenter, randomize, double-masked, parallel-group trial	6 weeks	OAG	BrBz-FC + PGA (88); PGA (94)	22.7 ± 2.1 mmHg; 22.4 ± 2.8 mmHg	Mean diurnal IOP change: BrBz-FC + PGA: −5.7 ± 0.3 mmHg (−24.7 ± 1.3%), PGA: −1.9 ± 0.3 mmHg (−8.2 ± 1.2%)	The incidence of ocular AEs: BrBz-FC + PGA: 35.5%, PGA: 21.1%; Blurred vision: BrBz-FC + PGA: 9.7%, PGA: 6.3%	Adding BrBz-FC to PGA therapy produced a mean diurnal IOP reduction of 5.7 mmHg (25%). BrBz-FC was superior PGA monotherapy, which was well-tolerated and no safety concerns

## 2 Methods

This review summarizes the effectiveness and safety of topical anti-glaucoma agents based on an overall review of related literatures published. Extensive references were obtained by searching keywords: prostaglandin analogs, rho-kinase inhibitors, β-adrenergic blocking agents, α-2 adrenergic agonists, carbonic anhydrase inhibitors, fixed-combination agents, glaucoma and so on. Systematic and large-scale clinical trials, meta-analysis and review articles were the main priority; because of the lacking of related articles, small-scale experiments and related cases were referenced. The main clinical trials more elaborated were summarize on Table 6.

## 3 Topical Monotherapy Agents

### 3.1 Prostaglandin Analogs (PGAs)

Prostaglandins (PGs) are potent biologically active metabolites of the arachidonic acid that modulate many biological responses in various tissues including the eye (Matsou and Anastasopoulos, 2018). PGAs represent the first-line medical therapy to cure POAG and OHT, since they target different PG receptors to reduce IOP (Diaconita et al., 2018; Angeli and Supuran, 2019) (Table 1).

#### 3.1.1 Prostaglandin F2-Alpha Receptor Agonists

Prostaglandin F2 (PGF2) analogs bind to PG receptors and promote muscle relaxation and extracellular matrix remodeling in the ciliary muscle and TM, thus increasing AH outflow in both the conventional and unconventional pathways (Matsou and Anastasopoulos, 2018; Jayanetti et al., 2020). PGF2 analogs reduce IOP by 25–33% (Diaconita et al., 2018), they are widely available on the market in most countries (Matsou and Anastasopoulos, 2018), and include latanoprost (Xalatan^®^), bimatoprost (Lumigan^®^), travoprost (Travatan Z^®^), and tafluprost (Tapros^®^) (Matsou and Anastasopoulos, 2018).

PGF2 analogs exert different IOP lowering effects, as demonstrated by clinical trials and meta-analysis: bimatoprost exerts the greatest IOP lowering effect and the highest incidence of hyperemia than other PGF2 analogs (Aptel and Denis, 2011; Yu and Welge-Lussen, 2013; Takagi et al., 2018). In addition, they are well tolerated and induce mild AEs including conjunctival hyperemia, increase in the eyelash growth, as well as changes in periocular pigmentation and iris color (Aptel and Denis, 2011; Yu and Welge-Lussen, 2013).

#### 3.1.2 Prostaglandin E2 Receptor Agonists

Prostaglandin E2 (PGE2) has high affinity for four different E-type prostaglandin (EP1-4) receptors (Matsou and Anastasopoulos, 2018). EP1-4 receptors are widely distributed in the human cornea, conjunctiva, TM, iris, ciliary body, and retina (Doucette and Walter, 2017; Matsou and Anastasopoulos, 2018). PGE2 receptor agonists increase uveoscleral outflow and induce morphological alterations in the tissues involved in the conventional pathway to decrease IOP by binding to EP1-4 receptors (Matsou and Anastasopoulos, 2018).

##### Prostaglandin E2 Receptor EP2 Subtype Agonists

###### Omidenepag Isopropyl (DE-117/G0G0H52U6K/STN-10117, Eybelis^®^)

####### Efficacy

Omidenepag isopropyl (OMDI) is a prodrug of omidenepag (Duggan, 2018) through the increase of both TM and uveoscleral outflow (Fuwa et al., 2018). The optimal dose of OMDI is 0.002% as demonstrated in the clinical trials (Aihara et al., 2019; Aihara et al., 2020a). IOP lowering effect of OMDI (0.002%) is not inferior to latanoprost (0.005%) (Aihara et al., 2019). The mean reduction of IOP after 4 weeks is 5.93 and 6.56 mmHg in the patients treated with OMDI (0.002%) and latanoprost (0.005%), respectively (Aihara et al., 2020a). In a phase 3 study, the percentage of the mean diurnal IOP reduction is 13.23% after 4 weeks of OMDI (0.002%) in patients who accepted latanoprost 0.005% with a reduction of IOP ≤15% during 8 weeks (Aihara et al., 2020b).

####### Safety and AEs

OMDI has better safety than latanoprost (Aihara et al., 2019). The ocular AEs induced by OMDI is mild or moderate, and the most frequently reported are conjunctival hyperemia, photophobia, and eye pain (Aihara et al., 2020a; Inoue et al., 2020a; Aihara et al., 2020b). Conjunctival hyperemia is dose-dependent (Aihara et al., 2019), and most frequently observed in small vessels (51%) in OMDI-treated eyes, and in both large and small vessels (81%) in ripasudil-treated eyes (Terao et al., 2020).

###### Aganepag Isopropyl (AGN-210961)

AGN-210961 has been formulated as a proprietary ophthalmic solution (Impagnatiello et al., 2019). Both AGN-210961 and bimatoprost 0.03% exert a similar IOP lowering effect at day 7 and week 4, as demonstrated by a clinical trial (NIH, 2014a). AGN-210961 do not exert any serious AEs, and the incidence of AEs in AGN-210961 group is also lower than bimatoprost group (NIH, 2014a).

###### AGN-210669

AGN-210669 is a sustained-release formulation of AGN-210961 (Impagnatiello et al., 2019) used in clinical trials for the treatment of POAG and OHT. But the safety and effectiveness of AGN-210669 is not superior to bimatoprost (NIH, 2013b; a). The combination of AGN-210669 (0.05%) and bimatoprost does not induce a significant change in the IOP lowering effect, and the AEs of the combination therapy also increased (NIH, 2013b; a). Therefore, AGN-210669 may not be a better medicine to control IOP.

##### Prostaglandin E2 Receptor EP3 Subtype Agonists

###### Sepetaprost (ONO-9054/DE-126)

####### Efficacy

The prodrug ONO-9054 is an isopropyl ester derivative of the biologically active free acid ONO-AG-367 in the treatment of glaucoma and OHT (Impagnatiello et al., 2019; Bhattaccharjee et al., 2020). ONO-9054 can rapidly be converted into ONO-AG-367 after ocular instillation and is characterized by dose-dependent systemic pharmacokinetics with rapid clearance (Suto et al., 2015; Harris et al., 2016).

A phase I clinical trial in OHT and POAG patients demonstrated that ONO-9054 reduces IOP in a dose-dependent manner and the greatest reduction is 29.6% at 9 h after the dose of ONO-9054 of 30 μg/ml on day 1 (Harris et al., 2016). In healthy volunteers, the mean reduction is 8.29 mmHg (28.2%) at 9 h after a dose of ONO-9054 of 30.0 μg/ml (Suto et al., 2015). The morning (AM) and evening (PM) dosage of 30 μg/ml ONO-9054 have similar sustained reduction in IOP, whereas the incidence of hyperemia and dryness is slightly increased in the patients subjected to the PM dose of ONO-0954 (Berlin et al., 2016). Miller Ellis, et al. observed that ONO-9054 (30 μg/ml) has a better IOP lowering effect than Xalatan once daily (Miller Ellis et al., 2017). The rate of the mean IOP reduction of ≤ −25%, ≤ −30%, and ≤ −35% in the ONO-9054-treated patients is 2.39, 2.37, and 4.85 times higher than the rate in the Xalatan-treated patients, and 2.4 times more patients achieve an IOP ≤15 mmHg than those treated with Xalatan (Miller Ellis et al., 2017).

####### Safety and AEs

ONO-9054 is well-tolerated and all AEs have a mild or moderate intensity (Miller Ellis et al., 2017). The AEs frequently reported are headache, anterior uveitis, vitreous detachment, conjunctival hyperemia, and blurred vision (Harris et al., 2016; Miller Ellis et al., 2017).

#### 3.1.3 Nitric Oxide-donating Prostaglandin F2-Alpha Analog

Nitric oxide (NO)-donating prostaglandin is composed of a prostaglandin F2 analogue moiety and NO-donating moiety, thus providing an IOP-lowering effect by two independent mechanisms (Impagnatiello et al., 2019). NO is an endogenous gas working as a signaling molecule inducing TM and Schlemm’s canal cell relaxation, decreasing cell volume, enlarging cell gap, and decreasing the outflow resistance through the activation of the soluble guanylyl cyclase/cyclic guanosine monophosphate (sGC-cGMP) signaling pathway (Krauss et al., 2011; Kaufman, 2017; Cavet and DeCory, 2018; Mao et al., 2020). In addition, it decreases AH production by inhibiting the Na^+^, K^+^-ATPase pump eventually lowering IOP (Wareham et al., 2018). NO also regulates the eye blood flow and protects the optic nerve by the sGC-cGMP signaling pathway (Wareham et al., 2018; Mao et al., 2020).

##### Latanoprostene Bunod (Vyzulta^®^)

###### Efficacy

LBN is the first NO-donating prostaglandin F2-alpha analog on the market (Addis and Miller-Ellis, 2018; Hoy, 2018a; Mehran et al., 2020). LBN administered every evening (QD) exerted a significantly greater effect and it is safer than timolol 0.5% (BID) (Kawase et al., 2016; Liu et al., 2016; Weinreb et al., 2016; Weinreb et al., 2018). The mean IOP is significantly lower in the LBN-treated patients (17.8–18.9 mmHg) than in those treated with timolol 0.5% (19.0–19.7 mmHg) at all time points (week 2, week 6, and month 3). A total of 22.9% patients treated with LBN achieved a mean IOP ≤18 mmHg, which is significantly more than that in those treated with timolol (11.3%) (Weinreb et al., 2016).

###### Safety and AEs

LBN is well-tolerated with no serious AEs and system AEs. Conjunctival hyperemia, eye irritation, and eye pain are the most reported ocular AEs (Medeiros et al., 2016). Blood pressure or heart rate does not change in correlation with LBN treatment (Kawase et al., 2016; Liu et al., 2016).

##### NCX 470

###### Efficacy

NCX-470 is a novel NO-donating bimatoprost with potentially greater IOP-lowering effect than bimatoprost monotherapy (Impagnatiello et al., 2015). A phase 2 clinical trial demonstrated that NCX-470 with well-tolerated was non-inferiority to latanoprost monotherapy (NIH, 2019a). The mean IOP reduction were 7.6–9.8 mmHg and 6.3–8.8 mmHg at day 28 in NCX-470 group and latanoprost group (NIH, 2019a).

###### Safety and AEs

Little evidence was found of treatment-related systemic effects or drug-related serious AEs associated to NCX-470. Conjunctival hyperemia was the most frequent AE in 16.8% of patients treated with NCX-470 (0.065%) *versus* 6.5% of patients treated with latanoprost (NIH, 2019a).

### 3.2 Rho-Kinase Inhibitors

The rho-kinases are serine/threonine kinase isoforms and effectors (Schehlein and Robin, 2019). Topical rho-kinase inhibitors inhibit rho-kinase isoforms in TM, thus directly increasing AH outflow in the TM pathway and consequently reducing IOP in a significant manner (Berrino and Supuran, 2019; Moura-Coelho et al., 2019). Rho-kinase inhibitors also exert a neuroprotective effect that in turn can exert a greater positive impact on the ocular blood flow, and even an antifibrotic effect that may be helpful if conventional glaucoma surgery is needed (Tanna and Johnson, 2018) (Table 2).

#### 3.2.1 Launched Rho-Kinase Inhibitors

##### Ripasudil (Glanatec^®^)

###### Efficacy

Ripasudil is the first rho-kinase inhibitor approved for the treatment of glaucoma (Berrino and Supuran, 2019). It promotes AH outflow by changing the TM cytoskeleton to reduce the outflow resistance and increasing the endothelial permeability of the Schlemm’s canal (Schehlein and Robin, 2019). Multiple clinical trials reported that ripasudil provides a great IOP lowering effect and exerts an additional reduction of IOP when used an add-on medication to PGAs and β-blockers (BBs) (Tanihara et al., 2013; Tanihara et al., 2016; Inoue and Tanihara, 2017). A phase 2 clinical study demonstrated that IOP-lowering effect of ripasudil is dose-dependent. When patients are treated with 0.1, 0.2, and 0.4% ripasudil for 8 weeks, the mean IOP reductions are 3.4 mmHg, 3.2 mmHg, and 3.5 mmHg, respectively (Tanihara et al., 2013).

###### Safety and AEs

Conjunctival hyperemia is the most frequent AE caused by ripasudil (Tanihara et al., 2013; Tanihara et al., 2016; Sakamoto et al., 2019) due to the relaxation of the smooth muscle in the blood vessels because of the inhibition of rho-kinase. However, this AE is mild, transient, and can resolve spontaneously in glaucoma patients (Sakamoto et al., 2019; Kusuhara and Nakamura, 2020). The other frequently observed AEs are blepharitis and allergic conjunctivitis (Tanihara et al., 2016).

##### Netarsudil (Rhopress^®^)

###### Efficacy

Netarsudil reduces the high IOP by inhibiting rho-kinase and norepinephrine transporter (Hoy, 2018b; Mehran et al., 2020). In this way, it increases TM outflow and reduces the episcleral venous pressure (Kazemi et al., 2018). Netarsudil 0.02% is well-tolerated and has a similar IOP lowering effect to timolol 0.5% twice daily (Kazemi et al., 2018; Serle et al., 2018; Khouri et al., 2019; Singh et al., 2020). Netarsudil once daily (PM) and twice daily have similar IOP lowering effect (Khouri et al., 2019). The mean IOP reduction at 3 months after netarsudil once daily and twice daily, as well as timolol twice daily is 16–21%, 22–24%, and 18–23% respectively (Serle et al., 2018).

###### Safety and AEs

Netarsudil causes non-serious AEs, generally mild in intensity (Kazemi et al., 2018; Serle et al., 2018; Kahook et al., 2019). The most frequent ocular AE is conjunctival hyperemia (Singh et al., 2020), followed by corneal deposits and conjunctival hemorrhage (Serle et al., 2018; Kahook et al., 2019). Other AEs include instillation site pain, blurred vision, increased lacrimation, reduction of visual acuity, eye pruritus, and erythema of the eyelid (Serle et al., 2018; Kahook et al., 2019).

#### 3.2.2. Clinical Rho-Kinase Inhibitors

##### RKI-983/SNJ-1656/Y-39983

SNJ-1656, also called Y-39983 or RKI-983, promotes the regeneration of the axon in damaged retinal ganglion cells (Yang et al., 2013). However, the IOP lowering effect is not very remarkable. In a phase I clinical trial, the mean IOP reductions are 2.2, 3.8, 4.3, and 4.0 mmHg before the instillation of the eyedrops in the morning and 1.5, 5.0, 4.4 and 4.5 mmHg at 2 h after the instillation in the morning in placebo and SNJ-1656 (0.03, 0.05 or 0.1%) group (Inoue et al., 2015). AEs are mild to moderate, and conjunctival hyperemia is the most frequently reported (Inoue et al., 2015).

##### AMA-0076/PHP-201

AMA0076, also called PHP-201, contains carboxylic ester moieties that allow the inactivation by esterase, resulting in an increased therapeutic window (Van de Velde et al., 2014). AMA0076 with a similar IOP-lowering efficacy as Y-39983 and latanoprost was demonstrated in the white rabbit experiment, and AMA0076 was more potent in preventing the IOP elevation in the acute hypertensive model (Van de Velde et al., 2014). However, no result of AMA-0076 on clinical trials was published.

##### Y-27632

Y-27632 is used in the treatment of OHT since it modulates the cytoskeletal alterations in TM cells to increase the conventional outflow, and relax the ciliary muscle contraction (Chen et al., 2020; Luo et al., 2021). At present, there is no related clinical study, but glaucomatous transgenic mice experiment demonstrated that Y-27632 can significantly decreased IOP and increased outflow facility, which greatly influenced the long-term IOP-lowering effect (Chen et al., 2020).

### 3.3 β-adrenergic Blocking Agents

BBs actively block the β-adrenergic receptors and their pharmacological properties depend on their various effects on the adrenergic signaling pathway (Bejan-Angoulvant and Angoulvant, 2020). The ocular AEs include conjunctival hyperemia, ocular surface discomfort, reduction in tear flow, and worsening of the dryness in the eye, while the systemic AEs include bradycardia, heart block and arrhythmia, bronchospasm, and worsening of underlying asthma or chronic obstructive pulmonary disease (Dikopf et al., 2017). These AEs are caused by the direct systemic absorption of BBs after ocular administration through the nasolacrimal system or the conjunctiva, without being subjected to first pass metabolism (Morales et al., 2016; Shukla et al., 2020). Although topical BBs is not associated with excess cardiovascular mortality as revealed in a meta-analysis with large population-based studies (Pinnock et al., 2016), ophthalmic BBs are not recommended in patients with asthma, chronic obstructive pulmonary disease, bradycardia, heart block, or uncontrolled heart failure (Marshall et al., 2018) (Table 3).

#### 3.3.1 Nonselective β-adrenergic Blocking Agents

Nonselective BBs reduce IOP by blocking β-adrenoceptor in the ciliary body and consequently decreasing AH production (Nocentini and Supuran, 2019). Timolol maleate (Timoptol^®^) is the first nonselective BB for the topical treatment of glaucoma and OHT (Sah and Suresh, 2017) and it reduces IOP by 27–35% (Nocentini and Supuran, 2019). Carteolol hydrochloride (Mikelan^®^) protects against light-induced oxidative stress in retina (Matsuo et al., 2019).

#### 3.3.2 Selective β-1 Adrenergic Blocking Agents

Betaxolol hydrochloride (Betoptic^®^) is a selective β1-blocker for the topical treatment of glaucoma, exerting an IOP reduction of 18–26% less than timolol (Dikopf et al., 2017).

#### 3.3.3 Selective β-2 Adrenergic Blocking Agents

##### Bamosiran (SYL040012)

###### Efficacy

SYL040012 targets the human β2-adrenergic receptor to inhibit its expression and consequently reduces the production of AH (Martinez et al., 2014; Jiang et al., 2021). It is also the first compound based on RNA interference (Moreno-Montanes et al., 2014). A phase I clinical trial demonstrated that no significant difference in IOP was found between the patients treated with 600 µg/eye once daily and those treated with 900 µg/eye twice daily (Moreno-Montanes et al., 2014). Patients in a phase 2 study were randomly divided to receive 80 µg (0.2%), 300 µg (0.75%), 900 µg (2.25%) SYL040012 or placebo once daily, and the results showed that 0.75% SYL040012 exerted a statistically significant reduction in IOP at day 14 (NIH, 2014c). More clinical studies are needed to explore the optimal dosing of SYL040012.

###### Safety and AEs

The AEs occur when SYL040012 is administered twice daily and include headache, conjunctival hyperemia, muscle spasm, unilateral stinging, bilateral itching, and difficulty in focusing. The diastolic blood pressure is significantly reduced only in patients treated with SYL0420012 at 600 µg/eye twice daily (Moreno-Montanes et al., 2014).

### 3.4 α-2 Adrenergic Agonists

α-adrenergic is divided into α1 and α2, which are the ones involved in the regulation of IOP. Ocular α2-adrenoreceptor induces conjunctival vasoconstriction, increases interpalpebral fissure, mydriasis, and decreases corneal oxygen tension (Cimolai, 2020). Brimonidine tartrate reduces AH production and increases the flow in TM and uveoscleral pathways (Nocentini and Supuran, 2019; Cimolai, 2020). Brimonidine tartrate (Alphagan^®^) is often administered as second line therapy or combined with other topical medications for the topical treatment of glaucoma and OHT (Hopf et al., 2020) resulting in an IOP reduction of 20–27% (Nocentini and Supuran, 2019; Cimolai, 2020). Brimonidine also offers a neuroprotective effect (Zhou et al., 2019; Conti et al., 2021). The significant AEs include blepharitis, blepharoconjunctivitis, conjunctivitis, conjunctival follicles, mild hyperemia, staining of the cornea, blurred vision, and foreign body sensation (Dikopf et al., 2017; Nocentini and Supuran, 2019) (Table 4).

### 3.5 Carbonic Anhydrase Inhibitors

Human carbonic anhydrase (hCA) is present in all living organism and it catalyzes carbon dioxide hydration that is regulating many physiological processes (Supuran et al., 2019; Supuran, 2020). hCA inhibitors exert IOP lowering effect by decreasing bicarbonate and AH secretion through the inhibition of hCA II, IV, and XII isoforms.

(Supuran et al., 2019; Ghorai et al., 2020). The first generation CAs inhibitors are sulfonamide hCA-II inhibitors including acetazolamide, methazolamide, and dichlorophenamide, which decrease IOP by 25–30% (Scozzafava and Supuran, 2014; Ghorai et al., 2020). They have been used as systemic antiglaucoma drugs for more than 50 years, but certain undesirable AEs are reported due to the inhibition of the enzymes present in the tissues than the ones in the eye (Carradori et al., 2015). The second generation of CAs inhibitors topically administered are sulfonamides, such as dorzolamide hydrochloride (Trusopt^®^) and brinzolamide (Azopt^®^), whose IOP lowering effect is comparable with the one of BBs, but less effective than PGAs (Eichhorn, 2013). Trusopt^®^ and Azopt^®^ are characterized by a better tolerance and less AEs compared to the first generation inhibitors, and the common reported AEs include stinging, burning or reddening of the eye, blurred vision, pruritus and bitter taste (Scozzafava and Supuran, 2014) (Table 4).

### 3.6 Adenosine Receptor Agonists

Adenosine is an essential component of the energy production and utilization systems of the body by providing the energetics necessary for the muscle movements, heartbeat, nerve signals, and chemical reactions (Jamwal et al., 2019). It exerts its functions by activating four adenosine receptor (AR) subtypes (A1, A2A, A2B, and A3) (Ciancetta and Jacobson, 2017). Adenosine is neuroprotective against excitotoxicity and metabolic dysfunctions that may be present in neurological and ocular diseases (Jamwal et al., 2019) (Table 4).

#### 3.6.1 Trabodenoson (INO-8875)

##### Efficacy

Trabodenoson is an adenosine mimetic that selectively acts on the A1 receptor subtype (Qiu, 2021). Trabodenoson can involve in matrix metalloproteinase signaling pathway associated with glaucoma (Qiu, 2021), consequently increasing TM outflow and exerting IOP lowering effect (Li et al., 2018). Trabodenoson exerts a dose-dependent and time-dependent IOP lowering effect (Myers et al., 2016). When patients receive of trabodenoson topical administration at the dose of 50, 100, 200, or 500 μg twice daily exerts a mean IOP reduction at day 14 to 1, 1.67, 2.5, and 3.2 mmHg, respectively, and a reduction to 4.1 mmHg with the dose of 500 μg at day 28 (Myers et al., 2016).

##### Safety and AEs

Trabodenoson has a good safety profile (Laties et al., 2016; Myers et al., 2016). The common systematic AEs such as headache, eye pain, back pain, dermatitis, and excoriation, were more frequently observed in the placebo-treated patients than in the trabodenoson-treated patients (Laties et al., 2016). Ocular AEs are uncommon and not serious, present for not longer than 24 h, and they are self-limited, and are usually mild in intensity (Laties et al., 2016).

## 4 Topical Fixed Combination of Two Agents

### 4.1 Fixed Combination of Prostaglandin F2a Analogs and β-blockers

#### 4.1.1 Fixed Combination Latanoprost and Timolol (Xalacom^®^)

##### Efficacy

LT-FC is currently one of the most widely used topical anti-glaucoma fixed-combination eye drops, which is usually chosen to cure patients with POAG and OHT who are insufficiently responsive to BBs, PGs, or other IOP lowering agents (Konstas et al., 2013c). LT-FC exerts a superior IOP lowering effect than the monotherapy using latanoprost or timolol. Mean IOP at 12 weeks is reduced to 10.2 mmHg in the LT-FC-treated patients, 8.9 mmHg in the latanoprost-treated patients and 7.2 mmHg in the timolol-treated patients and 73.5, 57.5, and 32.8% patients achieve a diurnal IOP reduction over 30% (Higginbotham et al., 2010). Zhao, et al. reported the IOP lowering effect of LT-FC versus non-fixed combination of latanoprost and timolol (LT-nFC), and their results revealed that LT-FC is noninferiority to LT-nFC regarding the mean diurnal IOP reduction after 8 weeks (8.6 *versus* 8.9 mmHg) (Zhao et al., 2011). Importantly, LT-FC shows a better compliance than LT-nFC (Zhao et al., 2011).

##### Safety and AEs

Various studies show similar AEs in patients treated with LT-FC, LT-nFC, latanoprost, and timolol monotherapies, and the most commonly reported one is conjunctival hyperemia (Higginbotham et al., 2010).

#### 4.1.2 Fixed Combination Bimatoprost and Timolol (Ganfort^®^)

##### Efficacy

BiT-FC has present in the market of more than 30 countries and regions worldwide OHT used for the reduction of IOP in patients with POAG (Fang et al., 2015). BiT-FC significantly decreases IOP in a greater extent than PGA, or timolol monotherapy (Lewis et al., 2010; Lequeu et al., 2013). BiT-FC also has better 24 h-IOP controlling effect than latanoprost monotherapy (Konstas et al., 2013b). The rates of the mean diurnal IOP reduction over 20% are 68.1, 58.1, and 38.0% in BiT-FC, bimatoprost, and timolol-treated patients, respectively, and the rates of IOP less than 18 mmHg are 23.3, 18.1, and 8.0%, respectively (Lewis et al., 2010). The mean 24-h IOP reduction was 12.2 mmHg (39.2%) in BiT-FC group, which was better than that in latanoprost group (9.9 mmHg, 31.9%) (Konstas et al., 2013b).

##### Safety and AEs

No addition AEs and serious AEs are reported in BiT-FC treatment group. Although, the incidence of AEs is more in the bimatoprost-treated patients (60.0%) than in the BiT-FC- (48.0%) and timolol-treated patients (31.6%), it is acceptable. Conjunctival hyperemia is the most frequently observed AE, with an incidence of 43.4% by bimatoprost, followed by 25.7% of BiT-FC and 8.7% of timolol (Fang et al., 2015).

#### 4.1.3 Fixed Combination of Travoprost and Timolol (DuoTrav^®^)

##### Efficacy

TrT-FC is preserved with benzalkonium chloride (BKC) and it is already introduced into the global market (Hoy et al., 2006; Konstas et al., 2012). Preservative-free (PF)-TrT-FC is one of the most recent PF agents that become available in Europe, which reduce the toxicity and improve the long-term health of the ocular surface in a better manner than TrT-FC (Denis, 2011; Konstas et al., 2012). A total of 162 patients in a prospective multicenter open-label study were treated with PGA monotherapy (travoprost, latanoprost, tafluprost, or bimatoprost) for over 3 months, then they switched to TrT-FC, and the observed reduction in IOP was 10.3 ± 12.7% (1.7 ± 3.1 mmHg), 9.4 ± 14.3% (1.6 ± 3.3 mmHg), and 10.1 ± 13.0% (1.7 ± 3.2 mmHg) after 4, 8, and 12 weeks, respectively (Nakano et al., 2015).

##### Safety and AEs

The most frequently observed AEs after TrT-FC treatment are ocular hyperemia, ocular discomfort, pruritus, and dryness (Hoy et al., 2006). Although, timolol has systemic side effects, no significant change from the baseline value of the mean systolic blood pressure is observed when patients switch to TrT-FC from PGA monotherapy (Nakano et al., 2015).

#### 4.1.4 Fixed Combination of Tafluprost and Timolol (Tapcom^®^)

##### Efficacy

PF-TfT-FC was used for the reduction of IOP in adults with OAG or OHT who require a combination therapy because they are insufficiently responsive to topical monotherapy with BBs or PGs (Hoy, 2015). PF-TfT-FC has a superior IOP lowering effect compared with agents used as monotherapy (Pfeiffer et al., 2014; Kaarniranta et al., 2016; Konstas et al., 2018), which is more suitable for evening administration (Konstas et al., 2018). In a phase 1 study, the result of the mean IOP was 10.3 mmHg for PF-TfT-FC, 10.9 mmHg for PF-tafluprost, and 11.1 mmHg for PF-timolol after 8 days in healthy volunteers (Kaarniranta et al., 2016). In another clinical trial, 189 patients treated with timolol who switched to PF-TfT-FC (n = 95) or PF-timolol (n = 94), showed a reduction of IOP to 7.1–9.0 mmHg in the PF-TfT-FC-treated patients and 6.5–8.1 mmHg in the timolol-treated patients and 375 patients treated with PGA who switched to PF-TfT-FC (n = 188) or PF-tafluprost (n = 187) for 3 months showed a reduction of IOP to 8.2–9.0 mmHg in the PF-TfT-FC-treated patients and 6.8–7.4 mmHg in the PF-tafluprost-treated patients (Pfeiffer et al., 2014).

##### Safety and AEs

The rate of AEs is more in the PF-TfT-FC-treated patients than in those treated with the single agent (Pfeiffer et al., 2014). Patients treated with PF-timolol show the lowest incidence (15.2%) of AEs in contrast to patients treated with PF-tafluprost (36.4%) and PF-TfT-FC (48.5%). The commonly reported ocular AEs related to the treatment with PF-TfT-FC include eye pain, eye pruritus, ocular hyperemia, and photophobia (Kaarniranta et al., 2016), while hyperemia is less common in patients treated with PF-TfT-FC than in those treated with PF-latanoprost (7.1% *vs* 21.4%) (Konstas et al., 2018).

#### 4.1.5 Fixed Combination of Latanoprost and Carteolol (Mikeluna^®^)

##### Efficacy

LC-FC has the same IOP lowering effect as the non-fixed combination of carteolol and latanoprost (LC-nFC) and LT-FC, and exerts a greater effect than carteolol and latanoprost monotherapy (Yamamoto et al., 2016; Inoue et al., 2018; Inoue et al., 2020b). In the study 1 of a two phase 3 clinical trial, 220 patients were treated with LC-FC (n = 113) or latanoprost (n = 116) for 8 weeks, while in the study 2, 175 patients were treated with LC-FC (n = 76), carteolol (n = 76), or LC-nFC (n = 37) for 8 weeks. The adjusted mean IOP reduction in the study 1 was 2.9 and 1.6 mmHg in the LC-FC and latanoprost-treated patients, in the study two was 3.5, 3.5, and 1.6 mmHg in LC-FC, LC-nFC, and carteolol-treated patients (Yamamoto et al., 2016). When patients with POAG or OHT were treated with LC-nFC and then switched to LC-FC, they showed an IOP of 15.0 ± 2.6, 15.1 ± 2.4, and 15.0 ± 2.4 mmHg at baseline, month 1 and 3, respectively (Inoue et al., 2018). When patients with POAG, NTG, or OHT were treated with LT-FC and then switched to LC-FC, they showed a not significantly changed mean IOP after 1 month (15.9 ± 3.1 mmHg) and 3 months (16.3 ± 3.8 mmHg) compared to the baseline IOP with LT-FC (16.1 ± 3.1 mmHg) (Inoue et al., 2020b).

##### Safety and AEs

The AEs related to LC-FC observed in clinical trials are mild (Yamamoto et al., 2016), and common ocular AEs include hyperemia, irritation, itching, pain, and blurred vision (Inoue et al., 2018). The incidence of ocular AEs decreases when the therapy switches from LC-nFC to LC-FC (Inoue et al., 2018), so LC-FC may provide better tolerance. The pulse rate and blood pressure do not significantly change in patients treated with LC-FC (Yamamoto et al., 2016; Inoue et al., 2018).

#### 4.1.6 Comparison of the Fixed Combination of Prostaglandin F2a Analogs and β-blockers

When comparing the efficacy of TrT-FC with that of LT-FC, the former showed a greater IOP lowering effect (Shoji et al., 2013; Konstas et al., 2014). Mean 24-h IOP reduction was 2.6 mmHg in patients treated with PF-TrT-FC and 2.2 mmHg in those treated with LT-FC (Konstas et al., 2014). Mean reduction in IOP from the baseline (14.8 ± 3.3 mmHg) at 12 weeks is significantly greater in patients treated with TrT-FC (2.4 ± 2.3 mmHg) than in those treated with LT-FC (1.1 ± 2.3 mmHg) (Shoji et al., 2013). No significant difference in ocular or systemic AEs is observed between TrT-FC- and LT-FC-treated patients, but stinging is significantly more in patients treated with LT-FC (19%) than in those treated with latanoprost (4.8%) (Konstas et al., 2014). A comparative study between BiT-FC and LT-FC discovered a significant reduction in IOP from the baseline without any change of the anterior ocular parameters. The biggest difference in IOP between treated and untreated eyes was 1.67 mmHg (13.6%) at 8 h after the instillation of BiT-FC and 1.93 mmHg (17.8%) at 10 h after the instillation of LT-FC. Conjunctival hyperemia is the most frequent AE in BiT-FC- and LT-FC-treated patients (33.3% *versus* 25.0%) (Shim et al., 2014). A comparative study showed that patients treated with BiT-FC have a lower IOP than those treated with TrT-FC and the mean IOP reduction is 11.17 and 7.89 mmHg after 6 months, respectively (Macky, 2014). However, in another clinical trial in which patients whose IOP were uncontrolled on BiT-FC accepted TrT-FC showed a decrease of the mean IOP by additional 16.5% at week 8 and 69.2% patients reached the target IOP (≤18 mmHg) (Scherzer et al., 2011). The common AEs are similar in BiT-FC- and TrT-FC-treated patients and include ocular hyperemia, ocular burning, blurred vision, foreign body sensation, and allergic reaction, while stinging/burning is less severe in patients treated with TrT-FC (Scherzer et al., 2011). PF-TfT-FC is as effective as LT-FC in terms of IOP-reducing effect, and induces fewer AEs such as eye irritation and eye pain, which were significantly reduced in patients using PF-TfT-FC (Suzuki et al., 2018). Patients in a phase IV study who switched from BiT-FC to PF-TfT-FC for 12 weeks showed an IOP after the treatment with PF-TfT-FC that was clinically insignificant and statistically non-inferior compared with BiT-FC (0.34 mmHg difference). However, the related ocular AEs including lacrimation, eye pruritus, and pruritus increase (Bourne et al., 2019). In a prospective, observer-masked, randomized study including 54 patients who received BiT-FC, LT-FC, or TrT-FC, a statistically significant reduction in diurnal/nocturnal IOP was observed with all medications, such as 4.6 ± 2.3 mmHg/3.2 ± 2.8 mmHg, 5.8 ± 2.4 mmHg/2.9 ± 1.9 mmHg, and 4.3 ± 1.7 mmHg/3.0 ± 1.6 mmHg, respectively, but no significant difference was observed among the three groups (Guven Yilmaz et al., 2018).

Overall, fixed combination and non-fixed combination of PGAs and β-blockers result in a similar IOP lowering effect, and both are superior to monotherapy, but fixed-combination medicines can significantly improve patient compliance and tolerence. Comparing to eye drops containing preservatives, preserver free medicines can better decrease the incidence of ocular side effects and increase ocular comfortable. So, it is necessary to modify the eye drops to be preservative-free to improve patient compliance and tolerance.

### 4.2. Fixed Combination of Prostaglandin F2a Analogs and Rho-Kinase Inhibitors

#### 4.2.1. Fixed Combination of Latanoprost and Netarsudil (Rocklatan^®^)

##### Efficacy

NL-FC is a glaucoma eye drop marketed as last and is recently approved by FDA for the treatment of OAG or OHT (Asrani et al., 2020). Multiple comparative studies observed that NL-FC had a superior IOP-lowering effect than netarsudil and latanoprost monotherapy (Asrani et al., 2019; Asrani et al., 2020). The percentage of IOP reduction observed in a phase 3 trial was 30.9–36.7% in patients treated with NL-FC compared to the 21.8–24.9% and 23.3–28.8% in patients treated with latanoprost or netarsudil monotherapy at month 3 (Asrani et al., 2019). Another phase 3 trial showed a mean diurnal IOP reduction to 7.8, 5.2, and 6.2 mmHg, respectively. NL-FC (32.3%) achieved a mean diurnal IOP ≤14 mmHg was 3 times higher than that observed after netarsudil (10.8%) and latanoprost (11.8%) monotherapy (Asrani et al., 2020).

##### Safety and AEs

NL-FC has a safety profile consistent with that of its individual components (Asrani et al., 2019). Conjunctival hyperemia is the most commonly reported in NL-FC group and netarsudil group (Asrani et al., 2020), which may be blood vessel smooth muscle relaxation and blood vessel dilation due to rho-kinase of calcium sensitization (Mehran et al., 2020).

### 4.3 Fixed Combination of Prostaglandin F2a Analogs and α-2 Agonists

#### 4.3.1 Fixed Combination of Bimatoprost and Brimonidine

##### Efficacy

BiBr-FC has been evaluated in a clinical trial for the treatment of glaucoma and OHT as an ophthalmic solution in 2013. IOP lowering effect of BiBr-FC was similar to the one of bimatoprost (0.01%) monotherapy and superior to the one of brimonidine (0.2%) monotherapy (NIH, 2015b).

##### Safety and AEs

The incidence of AEs is more in patients treated with BiBr-FC (71.05%) than in those treated with bimatoprost (69.44%) and brimonidine tartrate (52.63%) (NIH, 2015b), and common eye disorders include conjunctival hyperemia, eye pruritus, eye discharge, blepharospasm, ocular burning, punctate keratitis, eye pain, foreign body sensation, eye irritation, and blurred vision (Netland et al., 2003; NIH, 2015b). Conjunctival hyperemia and eye pruritus show significantly higher rates in BiBr-FC- and bimatoprost-treated patients than in those treated with brimonidine, while the other AEs are similar in the 3 groups (NIH, 2015b). The similar IOP lowering effect and higher AEs may limit the development of BiBr-FC.

### 4.4 Fixed Combination of Prostaglandin F2a Analogs and Carbonic Anhydrase Inhibitors

#### 4.4.1 Fixed Combination of Travoprost and Brinzolamide

##### Efficacy

TrBz-FC was used in phase III clinical studies for the treatment of OHT and OAG since 2014. However, both TrBz-FC and TrBz-nFC show no better IOP lowering effect than its marketed components (Travatan^®^ solution and Azopt^®^ suspension) at 8AM, 10AM, 12PM, 4PM, and 8PM after 6-weeks treatment (NIH, 2015a).

##### Safety and AEs

TrBz-FC is well-tolerated with no serious AEs and the reported AEs include ocular hyperemia, conjunctival hyperemia, eye irritation, eye pain, and dysgeusia (NIH, 2015a).

#### 4.4.2 Fixed Combination of Latanoprost and Dorzolamide

The fixed combination of latanoprost 0.005% and dorzolamide 2% was used in phase II clinical studies at Alleanza Pharmaceuticals for the treatment of glaucoma in 2013 (NIH, 2014b). The fixed combination of latanoprost (0.0025%) and dorzolamide (2%) will be completed in 2022 (NIH, 2020).

### 4.5 Fixed Combination of β-blockers and α-2 Adrenergic Agonists

#### 4.5.1 Fixed Combination of Brimonidine and Timolol (Combigan^®^, Aibeta^®^)

##### Efficacy

A fixed combination of brimonidine tartrate 0.2% and timolol 0.5% (Combigan^®^) was first launched by Canada in 2003. Another fixed combination of the ophthalmic solution composed of 0.1% brimonidine tartrate and 0.5% timolol (Aibeta^®^) was recently approved in Japan (Suzuki et al., 2020). Many clinical studies showed that BrT-FC had superior IOP lowering effect as well as effects in keeping it under control than their constituent agents (Spaeth et al., 2011; Kim et al., 2016), and it was noninferiority to latanoprost in reducing IOP (Katz et al., 2012). Katz, et al. found that 60.3% of patients treated with BrT-FC achieved diurnal IOP <18 mmHg compared to the 52.0% of patients treated with latanoprost after 12 weeks (Katz et al., 2012).

##### Safety and AEs

Tolerability and side effects were similar with the constituent parts without additional side effects. The most common local AEs of BrT-FC include eye irritation, dry eye, allergic reactions, lid erythema, lid edema, conjunctival follicles, corneal staining/erosion, and lens opacity (Katz et al., 2012; Kim et al., 2016). Systemic AEs in patients treated with BrT-FC included asthma (Kim et al., 2016).

### 4.6 Fixed Combination of β-blockers and Carbonic Anhydrase Inhibitors

#### 4.6.1 Fixed Combination of Timolol and Brinzolamide (Azarga^®^)

##### Efficacy

BzT-FC was launched in several European countries in 2009. It has the lowest daily cost and best effectiveness in China compared to other treatments (Xu et al., 2020). A prospective study demonstrated that bimatoprost and BzT-FC exert similar IOP lowering effects in patients with POAG at 8 weeks (Akyol et al., 2017). Importantly, BzT-FC maybe an appropriate choice for patients after uneventful phacoemulsification cataract surgery using Viscoat and Provisc, because BzT-FC can significant prevents IOP increase during the first 24 h after surgery (Georgakopoulos et al., 2013). In a clinical trial, when patients are treated with BzT-FC immediately after surgery or do not receive any ocular hypotensive medication, IOP changes from a preoperative value of +6.7 ± 2.98, +5.3 ± 3.26, and +1.4 ± 2.46 mmHg at 6, 12, and 24 h, to a postoperative value of −0.3 ± 2.95, +0.23 ± 3.49, and −1.76 ± 2.83 mmHg (Georgakopoulos et al., 2013).

##### Safety and AEs

BzT-FC is well tolerated (Rossi et al., 2011), although pulse rate and systolic blood pressure significantly change (Dixit et al., 2020). The frequently observed AEs are blurred vision, eye irritation, eye pain, and foreign body sensation (Sezgin Akcay et al., 2013; Alezzandrini et al., 2014; Aihara et al., 2017).

#### 4.6.2 Fixed Combination of Timolol and Dorzolamide (Cosopt^®^)

##### Efficacy

DzT-FC (fixed combination of Timolol 0.5% and Dorzolamide 2%) was the first IOP-lowering fixed combination approved by the US Food and Drug Administration (FDA) in the treatment of POAG and NTG (Konstas et al., 2021). PF-DzT-FC_1_ (preservative-free fixed combination of Timolol 0.5% and Dorzolamide 2%) and PF-DzT-FC_2_ (preservative-free fixed combination of Timolol 0.5% and Dorzolamide 1%) have also been launched in the market in recent year. In an open-label 2-center study, patients with NTG received DzT-FC for 12 weeks, and IOP was reduced by 21.7% at the peak drug effect and by 23.9% at 8 h after drug administration (Kim et al., 2014). DzT-FC has the same efficacy as latanoprost monotherapy in newly diagnosed NTG patients, and the difference of IOP reduction was 0.39 mmHg (Lee et al., 2016b). Visual-related quality of life and the Glaucoma Symptom Scale were significantly improved at week 8 when patients switched from DzT-FC to PF-DzT-FC_1_ (Abegao Pinto et al., 2014), and PF-DzT-FC_1_ showed a mean IOP reduction of 6.3% more than DzT-FC after 12 weeks (Renieri et al., 2010). Therefore, preservative-free DzT-FC may be a better choice than preservative DzT-FC.

##### Safety and AEs

DzT-FC does not exert systemic AEs and the most frequent ocular AEs are eye irritation and ocular hyperemia (Kim et al., 2014; Lee et al., 2016b; a). PF-DzT-FC_1_ improves local tolerability compared to DzT-FC (Renieri et al., 2010; Shedden et al., 2010).

#### 4.6.3 Comparison of the Fixed Combination of β-blockers and Carbonic Anhydrase Inhibitors

Several clinical trials demonstrated that BzT-FC exerts a better IOP-lowering effect (Rossi et al., 2011; Sezgin Akcay et al., 2013). IOP reduction was 6.42–9.74 mmHg (26.09–37.46%) and 8.16–12.41 mmHg (31.19–41.44%), respectively (Sezgin Akcay et al., 2013). But, IOP change from the baseline at 9 AM/11 AM pooled after 8 weeks was similar between BzT-FC (3.3/3.3 mmHg) and DzT-FC (2.9/3.4 mmHg) group (Aihara et al., 2017).

The most common ocular AEs include blurred vision, eye irritation, eye pain, and foreign body sensation (Sezgin Akcay et al., 2013; Aihara et al., 2017). Patients treated with DzT-FC have a significantly higher incidence of ocular irritation (33.3% *versus* 7.01%), ocular pain (29.8% *versus* 3.5%), and foreign body sensation (28.07% *versus* 5.2%) than those treated with BzT-FC, whereas blurred vision was reported more in patients treated with BzT-FC than in those treated with DzT-FC (14% *versus* 10.5%) (Sezgin Akcay et al., 2013; Aihara et al., 2017).

Generally, both fixed-combination medications represent highly effective without clear superiority of one agent over another; however, patients show more ocular comfortable in BzT-FC group. Comparing to DzT-FC, PF-DzT-FC significantly improved effectiveness and safety, which will be equal to or better than BzT-FC.

### 4.7 Fixed Combination of α-2 Adrenergic Agonists and Carbonic Anhydrase Inhibitors

#### 4.7.1 Fixed Combination of Brinzolamide and Brimonidine (Simbrinza^®^)

##### Efficacy

BzBr-FC was approved in United States and EU for the reduction of high IOP in patients with OAG and OHT, and it is the fixed-combination ophthalmic suspension to treat glaucoma available without timolol (Greig and Deeks, 2015). BzBr-FC induces a significantly lower mean IOP and exerts a more IOP reduction than either brinzolamide or brimonidine monotherapy (Katz et al., 2013; Nguyen et al., 2013; Realini et al., 2013; Aung et al., 2014). Importantly, BzBr-FC can significant lower IOP in patients after phacoemulsification cataract surgery. The mean change in postoperative IOP at 6, 12, and 24 h was −0.12, −1.12, and −1.89 mmHg in patients treated with BzBr-FC versus +3.85, +3.46, and +0.85 mmHg in patients of the control group (Georgakopoulos et al., 2020). BzBr-FC 3 times daily exerts a significantly more IOP reduction during the nocturnal period and the entire 24-h period compared to timolol 0.05% twice daily, and IOP lowering effect during the diurnal period is similar in both treatments (Seibold et al., 2017).

##### Safety and AEs

BzBr-FC is security with no serious AEs and other safety variables appeared (Katz et al., 2013), which also has no significant changes in visual acuity, anterior or posterior segment examination, pachymetry, and perimetry are observed (Realini et al., 2013). The most common AEs are blurred vision, dysgeusia, ocular hyperemia, dry mouth, and eye allergy (Katz et al., 2013; Realini et al., 2013; Aung et al., 2014; Weinreb et al., 2019).

### 4.8 Fixed-Combination of α2-Adrenoceptor Agonists and Rho-Kinase Inhibitors

#### 4.8.1 Fixed-Concomitant Brimonidine/Ripasudil

BrRi-FC (K-232) has been used in phase III clinical trials for the treatment of POAG and OHT since 2020. At present, no new reports on this topic are available.

### 4.9 Comparison of Topical Fixed Combination of Two Agents

#### 4.9.1 Comparison of the Fixed Combination of Prostaglandin F2a Analogs and Timolol With Carbonic Anhydrase Inhibitors and Timolol

Several clinical trials demonstrated that the fixed combination of prostaglandin F2a analogs and timolol has a greater IOP lowering effect than the fixed combination of carbonic anhydrase inhibitors and timolol (Eren et al., 2012; Babic et al., 2013). The mean diurnal IOP/peak IOP reduction is greater after the use of LT-FC (8.79 mmHg/7.4 mmHg) than after DzT-FC (7.79 mmHg/5.19 mmHg) at week-6 (Eren et al., 2012). The incidence of reported AEs is statistically lower after LT-FC therapy (Eren et al., 2012). A total of 86.7% of patients who received TrT-FC show a IOP reduction>25% compared with the 76.9% of patients treated with DzT-FC after 3 months (Babic et al., 2013). The most frequent ocular AEs in patients treated with TrT-FC are hyperemia (50%), blurred vision, and pruritus (6.7%), but dry eye sensation (30.8%) and foreign body sensation (23.1%) are more reported in patients treated with DzT-FC (Babic et al., 2013).

Overall, the IOP lowering effect of fixed-combination prostaglandin F2a analogs and timolol is significantly superior to fixed-combination carbonic anhydrase inhibitors and timolol, and both of them have no difference in ocular AEs. But the price of fixed-combination carbonic anhydrase inhibitors and timolol is lower, and patient can accept easily.

#### 4.9.2 Comparison of the Fixed Combination of Prostaglandin F2a Analogs and Timolol With Prostaglandin F2a Analogs and Brimonidine

Both brimonidine tartrate (0.1%) and timolol maleate (0.5%) used as adjunctive therapies to PGAs have similar effective, and the difference is only 0.36 mmHg in IOP reduction (Mizoue et al., 2017). No unexpected AEs were reported during the study, and the highest AEs was conjunctival hyperemia (2.8%) in brimonidine-treated patients and pruritus (4%) in timolol-treated patients (Mizoue et al., 2017).

#### 4.9.3 Comparison of the Fixed Combination of Prostaglandin F2a Analogs and Timolol With Brimonidine and Timolol

When compared directly of LT-FC, BiT-FC, and BrT-FC, no significant difference among them is observed. The reduction in IOP is 6.8 mmHg (32.1%), 6.7 ± 2.8 mmHg (30.2%), and 10.6 mmHg (42.2%) in LT-FC, BiT-FC, and BrT-FC group, respectively (Yavas et al., 2013). Hommer A, et al. also found that LT-FC and BrT-FC are effective in an equal manner in reducing IOP and they have no effect on the blood flow of the optic nerve head and the velocity of the retrobulbar flow (Hommer et al., 2012).

#### 4.9.4 Comparison of the Fixed Combination of Carbonic Anhydrase Inhibitors and Timolol With Brimonidine and Timolol

When compared directly to BrT-FC, BzT-FC shows more effective and tolerated. Patients who switched from BrT-FC to BzT-FC show a mean IOP reduction from the baseline (on BrT-FC) to 3.6 mmHg (17.1%), and 55.3% of patients achieve an IOP <18 mmHg at week 8 (Alezzandrini et al., 2014). BzT-FC is also more effective than BrT-FC in reducing IOP after phacoemulsification surgery, and IOP value after surgery at day 3 and 5 is −1.1 ± 2.70 and −2.19 ± 2.46 mmHg in the BzT-FC-treated patients, and −1.48 ± 2.53 and −1.72 ± 2.64 mmHg in the BrT-FC-treated patients (Balsak et al., 2018).

Multiple clinical trials reported that BrT-FC is better than DzT-FC in reducing IOP, although no statistically significant difference is found (Gulkilik et al., 2011; Feke et al., 2013; Seymenoglu et al., 2015). The side-effect profile of BrT-FC is similar to that of DzT-FC. But the incidence of the burning feeling and foreign body sensation are significantly higher in patients treated with DzT-FC (43 and 28%, respectively) than in patients treated with BrT-FC (19 and 12%, respectively) (Gulkilik et al., 2011).

#### 4.9.5 Comparison of the Fixed Combination of Carbonic Anhydrase Inhibitors and Timolol With Carbonic Anhydrase Inhibitors and Brimonidine

A clinical trial demonstrated that BzBr-FC is an effective and safe alternative β-blocker free fixed combination. A significant difference in mean morning IOP reduction is observed between DzT-FC- (7.0 ± 2.8 mmHg) and BzBr-FC-treated patients (8.4 ± 1.9 mmHg) for 12 weeks, but mean afternoon IOP reduction had no significant difference (Kozobolis et al., 2017). The common reported AEs are similar after both the two treatments (Kozobolis et al., 2017).

## 5 Topical Non-fixed Combination of Two Agents

### 5.1 Non-fixed Combination of Prostaglandin F2a Analogs and α-2 Agonists

#### 5.1.1 Non-fixed Combination of Latanoprost and Brimonidine

##### Efficacy

An important adjunctive (two-medicine) therapy to reduce IOP is represented by the non-fixed combination therapy of brimonidine tartrate (0.1, 0.15, 0.2%) twice daily and latanoprost (0.005%) once daily (Stewart et al., 2004). A multicenter, open-label, prospective evaluation study demonstrated that the adjunctive therapy with brimonidine purite 0.15% lead to an additional mean IOP reduction from a baseline value of 5.8 mmHg (26%) at peak drug effect after the treatment with 0.005% latanoprost and 3.3 mmHg (15%) at trough drug effect after 1 month (Mundorf et al., 2007). LBr-nFC (brimonidine 0.2% and latanoprost 0.005%) exerted a mean IOP reduction to 9.0 mmHg (33.9%) compared to DzT-FC 6.5 mmHg (25.3%) at 12 weeks in patients with pigmentary and pseudoexfoliative glaucoma (Zabriskie and Netland, 2003). Importantly, brimonidine can act as a supplement to therapy also in prevention as a neuroprotective agent (Conti et al., 2021).

##### Safety and AEs

No serious AEs are reported, and the most common AEs are ocular allergy (n = 2; 4.7%) and foreign body sensation (n = 2; 4.7%) in LBr-nFC-treated patients (Mundorf et al., 2007). While patients treated with LBr-nFC have a higher rate of conjunctival hyperemia than LT-FC-treated patients (Stewart et al., 2004).

#### 5.1.2 Non-fixed Combination of Travoprost and Brimonidine

##### Efficacy

A comparative clinical trial showed that timolol 0.5% treatment is associated with a significantly greater reduction in IOP compared with brimonidine 0.2% when added to travoprost 0.004%. The mean IOP reduction is 3.9 mmHg for timolol and 2.3 mmHg for brimonidine and the IOP reduction is 20.2 and 13.4% on day 28, respectively (Reis et al., 2006).

##### Safety and AEs

No systemic AEs are reported, and slight occasional conjunctival hyperemia is the only AEs in TrBr-nFC-treated patients (Reis et al., 2006).

### 5.2 Non-fixed Combination of Prostaglandin F2a Analogs and Carbonic Anhydrase Inhibitors

#### 5.2.1 Non-fixed Combination of Latanoprost and Brinzolamide

##### Efficacy

Several randomized clinical trials reported that the non-fixed combination of latanoprost 0.005% and brinzolamide 1% exerts a better IOP reducing effect than latanoprost monotherapy (Shoji et al., 2005; Nakamoto and Yasuda, 2007; Nakano et al., 2016). A comparative study demonstrated that LBz-nFC provides a more sustained IOP lowering effect than LT-nFC. LBz-nFC reduces both daytime and nighttime IOP, whereas LT-nFC only reduced IOP during daytime, with little effect on the nighttime value (Liu et al., 2009).

##### Safety and AEs

LBz-nFC represents a safe and effective treatment with no serious AEs reported during the observation period (Shoji et al., 2005). No significant difference in corneal endothelial cell density, systolic, diastolic, mean blood pressures, and pulse rates is observed before and after LBz-nFC administration (Nakamoto and Yasuda, 2007; Miura et al., 2008).

#### 5.2.2 Non-fixed Combination of Bimatoprost and Dorzolamide

##### Efficacy

Non-fixed combination of 0.03% bimatoprost once in the morning and 2% dorzolamide twice daily in patients with POAG exerts an additional hypotensive effect and reduces vascular resistance in the ophthalmic artery compared to bimatoprost monotherapy (Stankiewicz et al., 2010; Stankiewicz et al., 2011). However, BiDz-nFC led to a significant IOP lowering effect only at 4:00 h time point and show a lower IOP fluctuation compared to bimatoprost (4.6 mmHg *versus* 6.0 mmHg) (Stankiewicz et al., 2010).

##### Safety and AEs

No serious AEs are reported during study and systemic hypotension is the most common AEs in patients with BiDz-nFC (Stankiewicz et al., 2010).

### 5.3 Non-fixed Combination of β-blockers and α2-Adrenoceptor Agonists

#### 5.3.1 Non-fixed Combination of Betaxolol and Brimonidine

##### Efficacy

Non-fixed combination of 0.2% brimonidine tartrate and 0.5% betaxolol twice per day exerts statistically greater IOP reduction than their respective monotherapies (Chi et al., 2013). A comparative clinical trial revealed that the decrease IOP rate is 13.8–21.2% in patients treated with betaxolol, 19.8%–25.5% in patients treated with brimonidine and 22.2–33.2% in patients treated with BeBr-nFC after 8-weeks treatment (Chi et al., 2013).

##### Safety and AEs

No systemic AEs or statistical significant difference are found among patients treated with BeBr-nFC and those treated with monotherapies (Chi et al., 2013). The reported AEs include ocular foreign bodies, irritation, dizziness, headache, fatigue, and dryness of the mouth and nose (Chi et al., 2013).

### 5.4 Non-fixed Combination of β-blockers and Carbonic Anhydrase Inhibitors

#### 5.4.1 Non-fixed Combination of Betaxolol and Brinzolamide

##### Efficacy

DzT-FC is more effective after 24 h exposure compared to the effect of the fixed combination of 0.5% betaxolol and 1% brinzolamide, and the rate of IOP reduction is 18–24% and 14–19% in DzT-FC and BeBz-nFC-treated patients, respectively (Brubaker et al., 2000).

##### Safety and AEs

Anticipated AEs of BeBz-nFC are consistent with the AEs after betaxolol and brinzolamide monetherapy, and the incidence of systemic AEs does not increase (Brubaker et al., 2000).

### 5.5 Non-fixed Combination of α-2 Agonists and Carbonic Anhydrase Inhibitors

#### 5.5.1 Non-fixed Combination of Brimonidine and Dorzolamide

##### Efficacy

A randomized, double-masked study demonstrated that the non-fixed combination of dorzolamide 2% three times per day and 0.2% brimonidine tartrate two times per day exerts a greater reduction of AH flow, and BrDz-nFC has better IOP lowering effect than dorzolamide alone and brimonidine alone (Ermis et al., 2002; Tsukamoto and Larsson, 2004). The reduction in AH flow is 28.2 ± 18.0%, 19.3 ± 22.0%, and 37.2 ± 20.6% in brimonidine, dorzolamide and BrDz-nFC-treated patients, respectively and IOP reduction is 11.6 ± 10.1%, 8.5 ± 14.1%, and 17.9 ± 16.5%, respectively (Ermis et al., 2002; Tsukamoto and Larsson, 2004). Oztürk F, et al. found that non-fixed combination of timolol maleate and dorzolamide (TDz-nFC) is more effective in lowering IOP than BrDz-nFC, and the mean IOP reduction is 6.8 and 5.6 mmHg, respectively, after 1 year of treatment (Ozturk et al., 2005).

##### Safety and AEs

The most common ocular and systemic AEs are mild and include stinging/burning, itching, conjunctival hyperemia, irritation, dry mouth, fatigue, and bitter taste. No serious AEs are reported (Ermis et al., 2002; Ozturk et al., 2005).

### 5.6 Non-fixed Combination of Prostaglandin F2a Analogs and Rho-Kinase Inhibitors

#### 5.6.1 Non-fixed Combination of Latanoprost and Ripasudil

##### Efficacy

A clinical trial showed that the lowering effect of IOP exerted by LRi-nFC is superior than that exerted by latanoprost monotherapy, and mean IOP reduction in LRi-nFC and latanoprost-treated patients is 2.2 and 1.8 mmHg respectively, before instillation (9 AM) and 3.2 and 1.8 mmHg respectively, after instillation (11 AM) (Tanihara et al., 2015).

##### Safety and AEs

No new AEs are observed when ripasudil is added to latanoprost compared to the old AEs exerted by latanoprost, and conjunctival hyperemia is the most frequent AE in patients treated with LRi-nFC and ripasudil (55.9% *vs* 8.7%) (Tanihara et al., 2015).

### 5.7 Non-fixed Combination of β-blockers and Rho-Kinase Inhibitors

#### 5.7.1 Non-fixed Combination of Timolol and Ripasudil

##### Efficacy

A clinical trial demonstrated that TRi-nFC leads to a more IOP reduction than timolol monotherapy at 9 AM (before instillation) (2.4 *versus* 1.5 mmHg) and 11 AM (after instillation) (2.9 and 1.3 mmHg) (Tanihara et al., 2015).

##### Safety and AEs

No new AEs are reported when ripasudil is combined with timolol compared to the old AEs exerted by timolol, and the most common AE is conjunctival hyperemia in patients treated with TRi-nFC with 65.4% compared to 5.8% in the timolol-treated patients (Tanihara et al., 2015).

### 5.8 Comparison of Topical Non-fixed Combination of Two Agents

Most clinical trials demonstrated that the non-fixed combination of prostaglandin F2a analogs and carbonic anhydrase inhibitors exerts a greater IOP-lowering effect than the non-fixed combination of prostaglandin F2a analogs and alpha-2 agonists, and both of them have similar systemic and ocular AEs (Konstas et al., 2005; Feldman et al., 2007). A 3-months randomized clinical trial demonstrated that 79 patients who were treated with brinzolamide and 84 patients who were treated with brimonidine twice-daily as adjunctive therapy to travoprost have a diurnal IOP reduction to 2.8 and 2.1 mmHg, and the reduction of diurnal IOP ≥15% is 40 and 27.8% in TrBz-nFC- and TrBr-nFC-treated patients, respectively (Feldman et al., 2007). Konstas, et al. reported that the mean diurnal IOP reduction is 2.2 mmHg in LDz-nFC-treated patients *versus* 2.1 mmHg in LBr-nFC-treated patients (Konstas et al., 2005). Patients treated with LDz-nFC and LBr-nFC had similar ocular and systemic AEs, while a statistically more incidence of conjunctival hyperemia and bitter taste is observed in patients treated with LDz-nFC (n = 9 and 8) than in those treated with LBr-nFC (n = 3 and 0) (Konstas et al., 2005).

## 6 Topical Fixed Combination of Three Agents

### 6.1 Fixed Combination of β-blockers, α-2 Adrenergic Agonists, and Carbonic Anhydrase Inhibitors

#### 6.1.1 Fixed Combination of Dorzolamide, Timolol, and Brimonidine (Krytantek Ofteno^®^)

##### Efficacy

TDzBr-FC has been launched in Mexico since 2007 for the treatment of glaucoma and OHT, since it decreases AH production (Nocentini and Supuran, 2019; Cimolai, 2020; Ghorai et al., 2020; Shukla et al., 2020; Supuran, 2020), and increases TM and the uveoscleral outflow (Nocentini and Supuran, 2019; Cimolai, 2020; Shukla et al., 2020). PRO-122 is a preservative-free TDzBr-FC (PF-TDzBr-FC) (NIH, 2019b).

However, several clinical studies demonstrated that IOP lowering effect of PRO-122 is not inferior to Krytantek Ofteno (NIH, 2019b). And a phase III comparative study demonstrated that the crossover between PRO-122 and TDzBr-FC does not affect IOP, and the mean IOP reduction is 0.11 mmHg difference from the baseline after 60 days (Gomez-Aguayo et al., 2018; NIH, 2019b). Furthermore, an investigator-masked, crossover study demonstrated that BiT-FC once daily exerts a greater IOP lowering effect than TDzBr-FC twice daily (Garcia-Lopez et al., 2014).

##### Safety and AEs

The incidence of AEs is similar between PF-TDzBr-FC and TDzBr-FC-treated patients, and ocular burning is the one with the highest incidence among the eye disorders in both groups (Gomez-Aguayo et al., 2018; NIH). The cup/disk ratio and visual field are not statistically different in BiT-FC and TDzBr-FC-treated patients (Garcia-Lopez et al., 2014).

### 6.2 Fixed Combination of Prostaglandin F2a Analogs, β-blockers, and α-2 Adrenergic Agonists

#### 6.2.1 Fixed Combination of Bimatoprost, Timolol, and Brimonidine

##### Efficacy

Two comparative clinical studies demonstrated that TBiBr-FC has clinically and statistically significantly superior IOP lowering effects than BrT-FC (Hartleben et al., 2017; Belfort et al., 2020). A phase 3 study conducted in Mexico and Colombia revealed that the mean IOP changes from baseline are 10.03 and 9.18 mmHg, and percentage of IOP ≤13 mmHg is 33.7 and 14.8% for TBiBr-FC and BrT-FC respectively, after 12 weeks (Hartleben et al., 2017). Another phase III study conducted in Brazil demonstrated that the mean IOP reduction at 12 weeks is 10.45 mmHg in patients treated with TBiBr-FC and 8.28 mmHg in those treated with BrT-FC. A total of 28.9, 36.8, and 71.1% of patients treated with TBiBr-FC achieved levels of IOP ≤13, ≤14, and ≤16 mmHg compared to 15.7, 22.9, and 55.4% in patients treated with BrT-FC (Belfort et al., 2020). TBiBr-FC maybe a new promising fixed-combination three agents for patients whose IOP cannot be inadequate by combination two agents, but more clinical trials are needed to verify the effectiveness and safety.

##### Safety and AEs

The most common AEs are ocular side effects and none are serious in TBiBr-FC and BrT-FC-treated patients (Hartleben et al., 2017; Belfort et al., 2020). The most common AEs include conjunctival hyperemia, eye irritation, allergic blepharitis, allergic conjunctivitis, and dry eye. Conjunctival hyperemia, eye irritation, and eye pruritus are statistically significant in patients treated with TBiBr-FC than those treated with BrT-FC (Hartleben et al., 2017; Belfort et al., 2020).

## 7 Topical Non-fixed Combination of Three Agents

Topical non-fixed combinations of three agents are usually composed of a fixed combination of two agents and a monotherapy agent, and they are used in patients whose IOP cannot be well controlled by combination two agents. Importantly, there is no a safe and reliable fixed-combination three agents in widespread use today.

### 7.1 Non-fixed Combination of Prostaglandin F2a Analogs, β-blockers, and α-2 Adrenergic Agonists

#### 7.1.1 Non-fixed Combination of Brimonidine, Timolol and Travoprost

##### Efficacy

A clinical trial demonstrated that BzT-FC plus travoprost 0.004% (TBzTr-nFC) has a greater efficacy in late afternoon and during night and better 24-h IOP control than the one exerted by TBrTr-nFC (Konstas et al., 2013a). The reduction of 24-h IOP is 3.0 mmHg (14%) exerted by TBzTr-nFC and 2.1 mmHg (10%) exerted by TBrTr-nFC. TBzTr-nFC decreases IOP better than TBrTr-nFC at all the 3 selected time points (18:00, 22:00, and 02:00), with differences ranging from 1.0 to 1.8 mmHg, and no difference is observed among the other 3 time points (06:00, 10:00, and 14:00) (Konstas et al., 2013a).

##### Safety and AEs

The ocular and systemic AEs are more frequently reported after the use of the non-fixed combination of three agents compared with travoprost monotherapy (Konstas et al., 2013a). Transient blurred vision occurred significantly more frequently in TBzTr-nFC-treated patients than in those treated with BzT-FC. The incidence of itchiness, hyperemia, and fatigue are more in the TBrTr-nFC-treated patients, and other AEs were similar in the two treatments (Konstas et al., 2013a).

#### 7.1.2 Non-fixed Combination of Brimonidine, Timolol and Latanoprost

##### Efficacy

A 12-weeks, randomized, multicenter study demonstrated that LTBr-nFC exerts a greater IOP lowering effect than LT-nFC in patients whose IOP is not adequately controlled by latanoprost 0.005% alone (Fechtner et al., 2011). The results showed an additional IOP reduction of 8.3 mmHg (35.5%) and 6.2 mmHg (27.0%) at 10 am, the incidence of IOP less than 18 mmHg at both peak and trough measurements is 59.6% *versus* 42.6%, and IOP reduction over 20% is 72.3 and 57.5% in LTBr-nFC and LT-nFC-treated patients, respectively (Fechtner et al., 2011).

##### Safety and AEs

No statistically significant difference in overall AEs is found between patients treated with LTBr-nFC and LT-nFC, and the most common AEs are ocular allergy in the patients treated with LTBr-nFC (3.9%) and punctate keratitis in those treated with LT-nFC (2.9%) (Fechtner et al., 2011).

### 7.2 Non-fixed Combination of Prostaglandin F2a Analogs, β-blockers, and Carbonic Anhydrase Inhibitors

#### 7.2.1 Non-fixed Combination of Brinzolamide, Timolol and Travoprost

##### Efficacy

Brinzolamide 1% twice daily plus TrT-FC (TBrTr-nFC) has a better IOP lowering effect than TrT-FC once daily (Hollo and Kothy, 2008; Goldberg et al., 2012). Goldberg I, et al. reported that brinzolamide reduces IOP by 1.1–1.5 mmHg (6–9%) when added to TrTFC, and an additional IOP reduction is observed at diurnal IOP, specifically at 08:00 and 16:00 o’clock (Goldberg et al., 2012). Another comparative study demonstrated that the average decrease of diurnal IOP is 6.2 mmHg (21.8%) after the use of travoprost, 9.3 mmHg (32.6%) after the use of TrT-FC, and 11.2 mmHg (39.3%) after the use of TBrTr-nFC (Hollo and Kothy, 2008).

##### Safety and AEs

No serious AEs are reported during the study (Hollo and Kothy, 2008) and common AEs including conjunctival hyperemia, eyelashes, cataract, blurring, superficial punctate keratitis, conjunctival redness, reduced visual acuity, and itching are no statistically different between patients treated with TBrTr-nFC and those treated with TrT-FC (Hollo and Kothy, 2008).

#### 7.2.2 Non-fixed Combination of Dorzolamide, Timolol and Latanoprost

##### Efficacy

Most clinical studies reported that triple non-fixed combination therapy of 2% dorzolamide, 0.5% timolol, and 0.002% latanoprost has a superior IOP lowering effect (Akman et al., 2005; Lesk et al., 2008; Hatanaka et al., 2010; Konstas et al., 2017). Konstas AG, et al. found that LTDz-nFC protects IOP from fluctuation and further more IOP reduction than latanoprost, DzT-FC, and LT-FC, with a mean 24 h-IOP of 22.1 mmHg, 19.9 mmHg, 19.5 mmHg, and 16.5 mmHg, and a IOP fluctuation of 4.7 mmHg, 4.4 mmHg, 4.1 mmHg, and 3.6 mmHg in latanoprost, DzT-FC, LT-FC, and LTDz-nFC-treated patients (Konstas et al., 2017). A 4-weeks, open-label controlled clinical trial demonstrated that the mean baseline diurnal IOP is 14.44 ± 3.03 mmHg in patients treated with LTDz-nFC, which was significantly lower than that in patients treated with latanoprost monotherapy (15.60 ± 3.09 mmHg) (Hatanaka et al., 2010). Another nonrandomized interventional study revealed that the mean IOP decrease is 6.3 and 5.8 mmHg in LTDz-nFC and DzT-FC-treated patients, respectively, and the rate of IOP reduction >20% is 66.4 and 52.9% respectively, after 12 weeks (Lesk et al., 2008). Akman A, et al. found that IOP lowering effect of latanoprost 0.005% and brimonidine 0.2% as adjunctive therapies to TDz-FC is comparable (Akman et al., 2005); the mean reduction of peak/trough IOP is 5.2/3.5 mmHg after LTDz-nFC treatment and 4.6/2.9 mmHg after TBrDz-nFC treatment, and the rate of the peak/trough IOP reduction over 15% is 77.1%/40 and 77.7%/41.7%, respectively (Akman et al., 2005).

##### Safety and AEs

LTDz-nFC provides a safety profile consistent with that of its individual components. The majority of AEs are mild or moderate (Lesk et al., 2008), and included burning/stinging, superficial punctate keratitis, watering, itchiness, conjunctivitis hyperemia, dry eye, ocular irritation, dry mouth, foreign body sensation, and blurred vision (Konstas et al., 2008).

#### 7.2.3 Non-fixed Combination of Dorzolamide, Timolol and Tafluprost

##### Efficacy

A comparative, crossover study demonstrated that PF-tafluprost 0.0015% plus DzT-FC was compared to PF-tafluprost and latanoprost monotherapies, and PF-TDzTf-nFC is statistically and clinically more effective than both the compounds used as monotherapy. The daytime/nighttime IOP is lower in PF-TDzTf-nFC-treated patients (17.0/17.6 mmHg) than in PF-tafluprost-treated patients (2.3/21.5 mmHg) and latanoprost monotherapy-treated patients (22.3/22.1 mmHg). However, PF-tafluprost (3.9 ± 1.3 mmHg) exerts a lower 24-h IOP fluctuation compared to PF-TDzTf-nFC (4.4 ± 2.3 mmHg) and latanoprost (4.6 ± 1.6 mmHg) (Konstas et al., 2017).

##### Safety and AEs

The rates of AEs in PF-TDzTf-nFC and latanoprost-treated patients are similar, and they are were more than those in PF-tafluprost-treated patients. Burning (6.9%), stinging (20.9%), and bitter taste (11.6%) are significantly more common in patients treated with PF-TDzTf-nFC than in those treated with PF-tafluprost monotherapy (Konstas et al., 2017).

#### 7.2.4 Comparison of Non-fixed Combination of Prostaglandin F2a Analogs, β-blockers, and Carbonic Anhydrase Inhibitors

When patients received brinzolamide 1% (2 times a day) or dorzolamide 1% (3 times a day) plus a fixed combination therapy of latanoprost and a BB, the mean IOP reduction is 1.9 and 1.8 mmHg at week 8, respectively (Tsukamoto et al., 2005). The common AEs observed in this study include ocular irritation and blurred vision. Dorzolamide causes more intense ocular irritation than brinzolamide (74% *vs* 16%), and no significant difference in blurred vision is observed between the two groups (52% *vs* 37%) (Tsukamoto et al., 2005).

Both brinzolamide and dorzolamide added to a fixed-combination latanoprost-β-blockers show a similar IOP lowering effect, but brinzolamide (2 times a day) presents a better patient compliance and ocular comfortable than dorzolamide (3 times a day), which is more meaningful for glaucoma patients.

### 7.3 Non-fixed Combination of Prostaglandin F2a Analogs, α-2 Adrenergic Agonists and Carbonic Anhydrase Inhibitors

#### 7.3.1 Non-fixed Combination of Brinzolamide, Brimonidine and Travoprost

##### Efficacy

A phase 4 clinical trial involving 233 patients randomly treated with BzBr-FC 3 times per day plus travoprost 0.004% once daily (BzBrTr-nFC) or vehicle plus travoprost 0.004% for 6 weeks revealed that the mean diurnal IOP change from travoprost-treated baseline is significant more in BzBrTr-nFC-treated patients (5.0 mmHg, 21.9%) than in travoprost-treated patients (2.0 mmHg, 8.8%) (Feldman et al., 2016).

##### Safety and AEs

The incidence of AEs is higher in patients treated with BzBrTr-nFC compared to those treated with travoprost (30.8% 14.7%), and the most common AE in BzBrTr-nFC group is conjunctival hyperemia (Feldman et al., 2016).

#### 7.3.2 Comparison of Non-fixed Combination of Prostaglandin F2a Analogs, α-2 Adrenergic Agonists, and Carbonic Anhydrase Inhibitors

Several clinical trials reported that BzBr-FC as an adjunct to PGA exerts an additional IOP lowering effect (Feldman et al., 2016; Topouzis et al., 2021). The percentage of mean IOP reduction is 24.7% in patients treated with BzBr-FC plus PGA and 8.2% in those treated with vehicle plus PGA at week 6 (Feldman et al., 2016). 60.0% of patients achieving the goal of IOP ≤18 mmHg after BzBr-FC plus PGA treatment versus 20.7% of patients treated with vehicle plus PGA (Topouzis et al., 2021).

AEs in BzBr-FC plus PGA-treated patients (23.2%) are higher than those in patients treated with PGA (4.3%) (Topouzis et al., 2021), but it’s perfectly acceptable. The highest incidence of ocular and non-ocular AEs occurring in patients treated with BzBr-FC plus PGA are ocular hyperemia (5.3%) and dry mouth (5.3%) (Topouzis et al., 2021). The other common AEs include eye irritation, eye pruritus, and ocular hyperemia (Topouzis et al., 2021).

## 8 Discussion and Opinion

Nowadays, multiple classes of topical eye drops are available for the treatment of glaucoma, but it is still a challenge to establish the most reasonable and optimal prescription for every patient because systematically extensive clinical studies on different classes of medications are still limited. In this review, the most recent articles on topical anti-hypertensive ophthalmic agents and their characteristics were summarized, which will provide a reference for glaucoma patient care. Monotherapy is usually used for initial treatment, which cannot usually reduce IOP to a physiologically level for long time. Therefore, combination multiple agents play a vital role for long-term control of IOP. Fixed combination and non-fixed combination of two agents results in similar IOP-lowering effect, which are also superior to monotherapy, and no significant difference in local and systematic AEs was observed among them. If combination two agents fail to reduce IOP sufficiently, the second drug can be replaced or a third medication can be added to the fixed combination, or a fixed-combination of three agents can be used. Now, fixed combination three agents, such as TDzBr-FC, cannot be widely available in various countries due to its obvious increase of AEs and no significant improvement in IOP, and TBiBr-FC is still being in clinical trial. Research and development of new, reliable, effective and safe fixed combination three agents is a trend of great importance. Netarsudil is a new launched Rho-kinase inhibitor, which provides a better neuroprotection effect superior to other eye drops. Rho-kinase inhibitors might be combined with β-blockers and be suitable for those who are not candidates for PGAs. Furthermore, another potential fixed combination including latanoprost, netarsudil and timolol, may provide better IOP lowering effect and patient compliance, lower preservative exposure and the incidence of side effects by targeting more mechanisms with once per day comparing to traditional marketed medicines.

The current review revealed that the use of a fixed combination of topical anti-hypertensive ophthalmic agents is recommended as early as possible in patients with glaucoma, to better control its development and decrease the damage of the optic nerve. Fixed combination agents not only provide a better effect than monotherapy but also result in a better convenience and tolerance, and less AEs and ocular discomfortable than non-fixed combination. Apart from the effect and safety, local availability and costs are also important factors to consider. Thus, fixed combination agents are a promising choice in the treatment of glaucoma and ocular hypertension.

## 9 Conclusion

Glaucoma is still one of the most common causes of irreversible vision loss, and the reduction and control of IOP are necessary to decrease the rate of visual field deterioration to treat glaucoma. Despite numerous surgical approaches and classes of IOP-lowering drugs available, glaucoma remains a challenging chronic disease. Many new emerging strategies including medicines and surgery are developed for the treatment of glaucoma by affecting different pathways, providing a new choice to patients and offering additivity and complementary to current therapeutics, although they need long-term rigorous verification of safety and efficacy. The current recent studies revealed that fixed combination therapy with reliable safety and effectiveness is significantly important for patients with glaucoma and effective on glaucoma, and it’s of great importance to develop fixed combination agents with a high effect for a better control of glaucoma.

## Glossary

IOP: intraocular pressure
TM: trabecular meshwork
NTG: normal tension glaucoma
POAG: primary open-angle glaucoma
AH: aqueous humor
OHT: ocular hypertension
AEs: adverse effects
PGs: prostaglandins
PGF2: prostaglandin F2
PGE2: prostaglandin E2
OMDI: omidenepag isopropyl
NO: nitric oxide
sGC-cGMP: soluble guanylyl cyclase/cyclic guanosine monophosphate
QD: every evening
LBN: latanoprostene bunod
BBs: β-blockers
hCA: human carbonic anhydrase
AR: adenosine receptor
LT-FC: fixed combination Latanoprost and Timolol
BiT-FC: fixed combination Bimatoprost and Timolol
TrT-FC: fixed combination of Travoprost and Timolol
BKC: benzalkonium chloride
PF: preservative-free
TfT-FC: fixed combination of Tafluprost and Timolol
LC-FC: fixed combination of Latanoprost and carteolol
LC-nFC: non-fixed combination of carteolol and latanoprost
NL-FC: fixed combination of Latanoprost and Netarsudil
BiBr-FC: fixed combination of Bimatoprost and Brimonidine
TrBz-FC: fixed combination of Travoprost and Brinzolamide
LDz-FC: fixed combination of Latanoprost and Dorzolamide
BrT-FC: fixed combination of Brimonidine and Timolol
BzT-FC: fixed combination of Timolol and Brinzolamide
DzT-FC: fixed combination of Timolol and Dorzolamide
BzBr-FC: fixed combination of Brinzolamide and Brimonidine
BrRi-FC: fixed-concomitant of Brimonidine and Ripasudil
LBr-nFC: non-fixed combination of Latanoprost and Brimonidine
TrBr-nFC: non-fixed combination of Travoprost and Brimonidine
LBz-nFC: non-fixed combination of Latanoprost and Brinzolamide
BiDz-nFC: non-fixed combination of Bimatoprost and Dorzolamide
BeBr-nFC: non-fixed combination of betaxolol and Brimonidine
BeBz-nFC: non-fixed combination of betaxolol and Brinzolamide
BrDz-nFC: non-fixed combination of Brimonidine and Dorzolamide
LRi-nFC: non-fixed combination of Latanoprost and Ripasudil
TRi-nFC: non-fixed combination of Timolol and Ripasudil
TDzBr-FC: fixed combination of Dorzolamide, Timolol, and Brimonidine
TBiBr-FC: fixed combination of Bimatoprost, Timolol, and Brimonidine
TBrTr-nFC: non-fixed combination of Brimonidine, Timolol and Travoprost
TBzTr-nFC: non-fixed combination of Brinzolamide, Timolol and Travoprost
LTBr-nFC: non-fixed combination of Brimonidine, Timolol and Latanoprost
LTDz-nFC: non-fixed combination of Dorzolamide, Timolol and Latanoprost
TDzTf-nFC: non-fixed combination of Dorzolamide, Timolol and Tafluprost.
